# A double-masked, sham-controlled trial of rose bengal photodynamic therapy for the treatment of fungal and acanthamoeba keratitis: Rose Bengal Electromagnetic Activation with Green Light for Infection Reduction (REAGIR) study

**DOI:** 10.1186/s13063-024-08376-3

**Published:** 2024-08-28

**Authors:** NV Prajna, P Lalitha, S Sharma, D de Freitas, A Höfling-Lima, N Varnado, S Abdelrahman, V Cavallino, BF Arnold, TM Lietman, J Rose-Nussbaumer

**Affiliations:** 1 Aravind Eye Hospital, Madurai, India; 2 Federal University of São Paulo, São Paulo, Brazil; 3 Francis I. Proctor Foundation, University of California, Sao Paulo, Brazil; 4 UCSF Epidemiology and Biostatistics, University of California, San Francisco, USA; 5 UCSF, Department of Ophthalmology, University of California, San Francisco, USA; 6 Byers Eye Institute, Stanford University, 2452 Watson Ct, Palo Alto, CA 94303 USA

**Keywords:** Infectious keratitis, Cornea, RB-PDT

## Abstract

**Background:**

Infectious keratitis secondary to fungus or acanthamoeba often has a poor outcome despite receiving the best available medical therapy. In vitro rose bengal photodynamic therapy (RB-PDT) appears to be effective against fungal and acanthamoeba isolates (Atalay HT et al., Curr Eye Res 43:1322–5, 2018, Arboleda A et al. Am J Ophthalmol 158:64-70, 2014). In one published series, RB-PDT reduced the need for therapeutic penetrating keratoplasty in severe bacterial, fungal, and acanthamoeba keratitis not responsive to medical therapy.

**Methods:**

This international, randomized, sham and placebo controlled 2-arm clinical trial randomizes patients with smear positive fungal and acanthamoeba and smear negative corneal ulcers in a 1:1 fashion to one of two treatment arms: 1) topical antimicrobial plus sham RB-PDT or 2) topical antimicrobial plus RB-PDT.

**Discussion:**

We anticipate that RB-PDT will improve best spectacle-corrected visual acuity and also reduce complications such as corneal perforation and the need for therapeutic penetrating keratoplasty. This study will comply with the NIH Data Sharing Policy and Policy on the Dissemination of NIH-Funded Clinical Trial Information and the Clinical Trials Registration and Results Information Submission rule. Our results will be disseminated via ClinicalTrials.gov website, meetings, and journal publications. Our data will also be available upon reasonable request.

**Trial registration:**

NCT, NCT05110001, Registered on November 5, 2021.

**Supplementary Information:**

The online version contains supplementary material available at 10.1186/s13063-024-08376-3.

## Key messages

Fungal or acanthamoeba keratitis often has a poor outcome despite receiving the best available medical therapy. Rose bengal photodynamic therapy (RB-PDT) may directly kill infectious organisms and strengthen the cornea, reducing the risk of perforation or need for therapeutic penetrating keratoplasty. This protocol describes the first large, NIH-funded, randomized clinical trial to study this therapy in patients and will determine whether RB-PDT is a beneficial adjunctive therapy for infectious keratitis.

**Supplementary Information:**

The online version contains supplementary material available at 10.1186/s13063-024-08376-3.

## Introduction

The photochemical reaction produced during cross-linking (CXL) may benefit patients with infectious corneal ulcers through direct anti-microbial effects as well as increased resistance of corneal tissue to enzymatic degradation [1–3]. Activation of a photosensitizer such as riboflavin with exposure to a specific wavelength of light results in release of reactive oxygen species and promotes chemical covalent bond formation between adjacent collagen molecules. CXL with riboflavin is currently used as a treatment for corneal ectatic disorders such as keratoconus and post-LASIK ectasia and has been shown to stiffen the cornea and allow it to retain its normal shape [4–7]. Recently, another similar treatment has been proposed that uses rose bengal (RB) as the photosensitizer and green light (532 nm) and is termed rose bengal photodynamic therapy (RB-PDT) [8]. RB-PDT appears to have similar effects on corneal biomechanical properties, is safe for limbal stem cells and endothelium, and demonstrates less toxicity to keratocytes in vitro than traditional CXL [9–14].

Reactive oxygen species are thought to have an antiseptic effect against a broad range of pathogens [15]. In vitro studies suggest that CXL is effective against common bacterial pathogens, including drug resistant organisms such as *Pseudomonas* and MRSA [16, 17]. In vitro studies have demonstrated limited benefit of CXL for fungal or acanthamoeba keratitis, and one randomized clinical trial also did not show a benefit of adjuvant CXL in filamentous fungal keratitis patients [18]. CXL for infectious keratitis is also identified in the literature as photoactivated chromophore for infectious keratitis (PACK-CXL) [19–21]. In vitro RB-PDT appears to be much more effective against fungal and acanthamoeba isolates [22, 23].

Infectious keratitis secondary to fungus or acanthamoeba often has a poor outcome despite receiving the best available medical therapy. For example, the NIH-funded MUTT II randomized controlled trial (RCT) of severe filamentous fungal keratitis demonstrated a 50% rate of full thickness corneal perforation or need for TPK despite maximal medical therapy, including topical natamycin, topical voriconazole, and oral voriconazole [24, 25]. Clinical studies have suggested a benefit of RB-PDT in infectious keratitis not responsive to medical therapy [26]. One published series demonstrated a 72% reduction in the need for TPK in severe non-responsive bacterial, fungal, and acanthamoeba keratitis after RB-PDT [27]. Here, we propose a randomized clinical trial to investigate adjunctive RB-PDT in the treatment of fungal, acanthamoeba, and smear- and culture-negative keratitis.

## Methods/design

### Study design

**Fig. 1:**
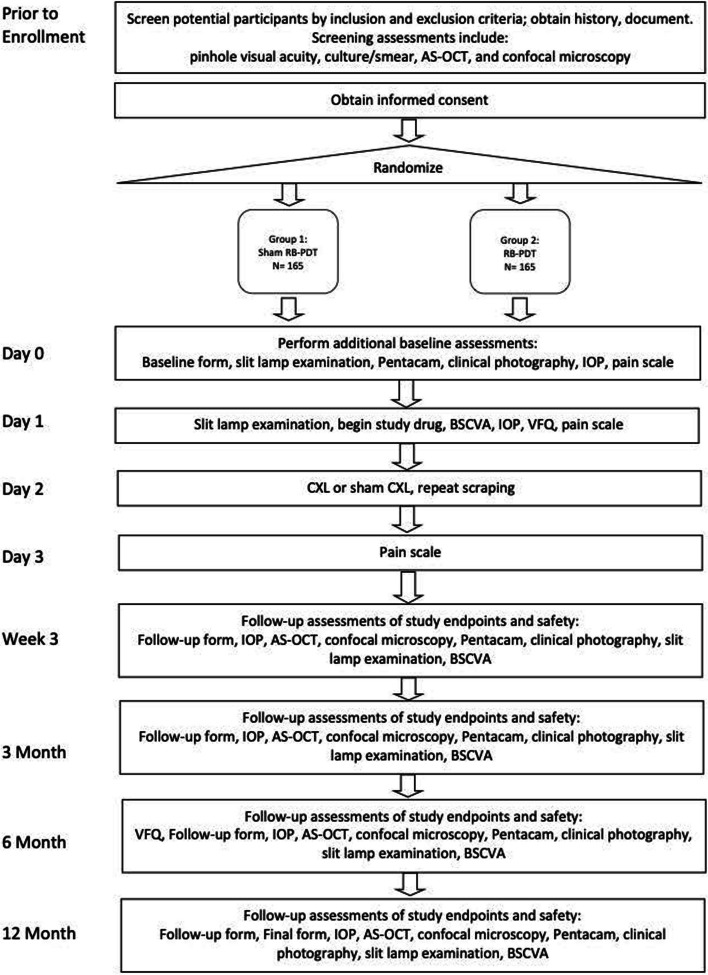
Schema of Rose Bengal Electromagnetic Activation with Green Light protocol

The Rose Bengal Electromagnetic Activation with Green Light for Infection Reduction (REAGIR) study is an international, randomized, outcome masked, sham-controlled 2-arm clinical trial (full protocol available as online supplement). The purpose of this study is to determine if 6-month visual acuity is superior with RB-PDT in addition to standard antimicrobial therapy versus standard therapy with antimicrobials and sham RB-PDT. Patients presenting to one of the Aravind Eye Hospitals in India or the University Hospital in São Paulo with smear and/or culture-positive fungal, acanthamoeba, or smear- and culture-negative keratitis and moderate vision loss, defined as Snellen visual acuity of 20/40 or worse and corneal thickness of greater than or equal to 350 μm as measured on AS-OCT, will be included. Figure 1 provides a schematic outline of the study.

Those who agree to participate will be randomized in a 1:1 fashion to one of two treatment arms:

- Group 1, sham RB-PDT: anti-microbial* plus sham RB-PDT
- Group 2, RB-PDT: anti-microbial* plus RB-PDT

* anti-microbials include moxifloxacin 0.5% for smear- and culture-negative keratitis, cationic antiseptic such as chlorhexidine gluconate 0.02% or polyhexamethylene biguanide 0.3% for acanthamoeba keratitis, and polyene macrolide such as natamycin 5% or amphotericin b 0.12% for fungal keratitis.

### Objective and hypothesis

The objective of this study is to determine if RB-PDT is a beneficial adjuvant in the treatment of filamentous fungal, acanthamoeba, or smear- and culture-negative keratitis. We anticipate that RB-PDT will result in better best spectacle corrected visual acuity (BSCVA) at 6 months compared with antimicrobial alone.

### Study oversight

An independent data and safety monitoring committee (DSMC) oversees the data collection and safety of the study. The DSMC members have expertise in ophthalmology with cornea subspecialty training, biostatistics, and ethics. Interim reports for the DSMC are prepared by the data coordinating center at the F.I. Proctor Foundation (Proctor) at UCSF. These reports include (a) recruitment overall and by study site, (b) compliance, and (c) retention. The reports also list study outcomes, including 6-month BSCVA and microbiological outcomes, and all adverse outcomes, including mortality and perforations or need for therapeutic penetrating keratoplasty (TPK). The DSMC meets annually in person and biannually via teleconference to monitor study progress and safety. There are also ad hoc meetings as needed. Study investigators conduct site visits at least biannually. The principal investigators notify the DSMC, study sites, and institutional review boards of any changes to study protocols or any deviations from the trial protocols.

### Setting

Participants will be enrolled at four sites in India and one site in Brazil. The study has obtained institutional review board approval at each facility and government approval in each country as well as at the University of California, San Francisco (IRB# 18–26,045). In India, participants will be enrolled at Aravind Eye Hospitals in Madurai, Coimbatore and Pondicherry (IRB# 2020009CLI ICMR# 011–26589492). In Brazil, participants will be enrolled at the University Hospital of São Paulo, Sao Paulo (IRB# 482/21 CONEP# 5.900.981). These sites were chosen because of the high volume of infectious keratitis cases seen and their ability to enroll study participants while adhering to study protocols and ensuring excellent follow-up.

### Inclusion and exclusion criteria

The inclusion criteria include age greater than 18 and presence of ulcer that is either smear and culture negative or is smear or culture positive for filamentous fungus or acanthamoeba. They must have a Snellen visual acuity of 20/40 or worse with a central corneal thickness greater than or equal to 350 microns as measured by anterior segment optical coherence tomography (AS-OCT). Exclusion criteria include evidence of concurrent viral keratitis, impending or frank corneal perforation, involvement of the sclera, non-infectious or autoimmune keratitis, history of recent intraocular surgery or prior corneal transplant, and fellow eye visual acuity worse than 20/200. The investigator will confirm their ability to understand the study and willingness to participate.

### Recruitment and retention strategy

Patients presenting to recruitment sites with smear-positive typical bacterial corneal ulcers, smear- or culture-positive fungal or acanthamoeba ulcers, or smear- or culture-negative ulcers with moderate to severe vision loss will be approached for possible inclusion in the study. For eligible patients, the study will be explained in the local language (Tamil at the Aravind Eye Clinics, English or Spanish at UCSF and University of Miami, and Portuguese at UNIFESP) in addition to the risks and benefits of participating in the study. Patients at some Aravind sites will be admitted to the hospital for the first 3 days of the study, ensuring minimal loss to follow-up through day 3. Patients will schedule their follow-up visits with the study coordinator while they are in inpatient care. The study coordinator will give the patient written documentation of their upcoming visits and will follow-up with a phone call as their appointments approach. Previous studies with Aravind and UCSF, including the original Steroids for Corneal Ulcers Trial, the Mycotic Ulcer Treatment Trials I and II, and the small cross-linking for bacterial keratitis feasibility assessment study we performed, have had high retention and leave us confident that this study will have high retention as well. In addition, regular monitoring and reports of follow-up by DCC and site visits by CCC will encourage excellent follow-up.

### Randomization

Each study eye is randomly assigned to the treatment group. Block randomization stratified by study site using randomly permuted block lengths was performed using a computer program (Statistical package R; Version 2.12; R Foundation for Statistical Computing, Vienna, Austria) by the data coordinating center. Once an eye is enrolled in the study, the study coordinator will assign the study participant’s eye an ID (alpha-numeric code), and topical antimicrobial will begin every hour for 2 days and then every 2 h while awake until resolution of the epithelial defect. The study coordinator will organize the procedure in the operating room within 48 h. Once the study participant has been assigned a study participant ID and randomized to treatment group, they will be included in the intent to treat analysis.

### Intervention and masking

Study participants will undergo RB-PDT or sham RB-PDT within 48 h of enrollment. Those randomized to the RB-PDT arm will receive a 30-min loading dose of topical 0.1% rose bengal drops applied in 5-min intervals to the de-epithelialized cornea. Full penetration through the cornea with anterior segment flare will be confirmed prior to CXL procedure. This will be followed by exposure to continuous 6 mW/cm^2^ custom-made green light LED source for 15 min (total of 5.4 J/cm^2^). During irradiation, patients will continue to receive topical rose bengal at 5-min intervals. Sham RB-PDT simulates this experience; however, a green light will be shined adjacent to the patient, careful to avoid exposure to the cornea, and the cornea will be covered with a corneal light shield. In place of rose bengal, we will use either saline drops. Rose bengal will not be used in the sham procedure due to concern that the photochemical activation of the rose bengal may occur with exposure to ambient light and therefore produce some treatment effect. All study participants will have repeat corneal cultures 30 min after the RB-PDT or sham RB-PDT procedure.

Due to the nature of the surgical intervention, the surgeon and technician performing cross-linking will not be masked. The patient, physician performing repeat scraping and clinical follow-up, microbiologist, and refractionist performing the BSCVA will be masked to treatment arm.

### Data collection and management

Data collection is the responsibility of the clinical trial staff at the site under the supervision of the site investigator. The investigator is responsible for ensuring the accuracy, completeness, legibility, and timeliness of the data reported. Table 1 outlines the schedule of enrolment, interventions, and assessments.

**Table 1:** Schedule of enrolment, interventions, and assessments for the Rose Bengal Electromagnetic Activation with Green Light for Infection Reduction trial

	Visit 1 Day 0	Visit 2 Day 1	Visit 3 Day 2	Visit 4 Day 3	Visit 5 3-week follow-up	Visit 6 3-month follow-up	Visit 7 6 month follow-up	Visit 8 12-month follow-up
Enrolment								
Consent and authorization	X							
Baseline form	X							
Clinical drawing	X	X			X	X	X	X
VFQ		X					X	
Follow-up form		X			X	X	X	X
Final form								X
Interventions								
CXL/sham CXL			X					
Study medication^b^		X						
Assessments								
IOP	X	X			X	X	X	X
Pain scale	X	X		X				
AS-OCT	X				X	X	X	X
Confocal microscopy	X				X	X	X	X
Pentacam topography	X				X	X	X	X
Clinical photography^a^	X				X	X	X	X
Slit lamp examination	X	X			X	X	X	X
BSCVA/ETDRS/MRx		X			X	X	X	X
Pinhole visual acuity	X							
Culture/smear	X		X					
Total visit time	2 h	2 h	3 h	0.5 h	1 h	1 h	1 h	1 h

^a^Clinical photographs also taken upon adverse events

^b^Difluprednate versus placebo starting at 24 h

Clinical data (including adverse events (AEs), concomitant medications, and expected adverse reactions data) and clinical laboratory data will be entered into Research Electronic Data Capture (REDCap), a 21 CFR Part 11-compliant data capture system provided by the data coordinating center at UCSF. These data will be kept confidential. The data system includes password protection and internal quality checks, such as automatic range checks, to identify data that appear inconsistent, incomplete, or inaccurate.

The trial steering committee is made up of members of both the clinical coordinating center (CCC) and the data coordinating center (DCC). The committee will meet weekly to monitor trial progress. Clinical site monitoring is conducted by the CCC at Stanford University to ensure that the rights and well-being of trial participants are protected, that the reported trial data are accurate, complete, and verifiable, and that the conduct of the trial is in compliance with the currently approved protocol/amendment(s), with International Conference on Harmonization Good Clinical Practice (ICH GCP), and with applicable regulatory requirement(s). The data coordinating center will conduct regular weekly off-site reviews of data entered in REDCap to ensure 100% data verification and prepare progress reports for the CCC as well as for the National Institute of Health appointed Data and Safety Monitoring Committee (DSMC).

Each clinical site will perform internal quality management of study conduct, data and biological specimen collection, documentation, and completion. It is the responsibility of the local site investigator to report deviations and serious adverse events to the medical monitor, CCC, and DCC. Protocol deviations must be sent to the reviewing Institutional Review Board (IRB) per their policies. The site investigator is responsible for knowing and adhering to the reviewing IRB requirements.

### Primary outcome measurement and statistical analyses

#### Visual acuity

The primary outcome will be 6-month best spectacle-corrected visual acuity (BSCVA). BSCVA will be measured in a masked fashion using the EDTRS chart with the patient seated 4 m away, and the room lights will be set between 50 to 100 foot-candles. We will use multiple linear regression models to evaluate BSCVA measured with covariates for treatment arm, study site (randomization strata), and baseline pinhole visual acuity.

### Secondary outcome measures and statistical analyses

#### Visual acuity at additional time points

As secondary analyses, we will also look at 3-week, 3-month, and 12-month BSCVA. We will use multiple linear regression models to evaluate BSCVA measured with covariates for treatment arm, study site (randomization strata), and baseline pinhole visual acuity. A number of subgroup analyses will be performed including organism subtype, infiltrate and/or scar location, and prior antimicrobial use.

#### Microbiological cure

Studies have suggested that in addition to providing an initial diagnosis, repeated culture can be used to assess response to treatment and is highly correlated with clinical outcomes such as visual acuity [28–31]. We will re-culture all study participants at day 2 to assess the effect of RB-PDT on rate of microbiological cure. We hypothesize that those in the RB-PDT group will have a higher rate of microbiological cure on day 2 cultures than those randomized to sham RB-PDT.

We propose the primary analysis to be a Fisher’s exact test comparing the proportion of positivity at follow-up between initially culture-positive individuals who were assigned to RB-PDT versus initially culture-positive individuals assigned to sham RB-PDT. Additionally, we will report the results for initially culture-negative individuals as a supplementary analysis in a logistic regression with assignment, indicators for site (randomization strata), and initial culture results as covariates.

#### Scar/infiltrate

Infiltrate and/or scar size will be measured at the slit lamp by a masked physician by taking the geometric mean of the longest diameter and longest perpendicular to that diameter in millimeters. Hypopyon height will also be recorded in millimeters at the slit lamp. The analysis for scar and/or infiltrate size will follow the templates for visual acuity given above. Multiple linear regression models will be used to evaluate 12-month scar size by treatment arm while correcting for baseline measurements. Corneal thinning and scarring will be evaluated similarly using anterior segment optical coherence tomography (AS-OCT) correcting for baseline values.

#### Visual Function Questionnaire (VFQ)

VFQ will be compared between arms controlling for day 1 VFQ. The Brazilian version of the NEI-VFQ will be used in Sao Paulo, and the Indian-VFQ (IND-VFQ) will be used in India. This will be conducted using linear regression with baseline and assignment variables.

#### Pentacam scheimpflug tomography

Pentacam is a rotating Scheimpflug camera, which provides 3-dimensional images of the cornea. In addition to topographic maps with keratometric readings of the anterior and posterior cornea, Pentacam reports on the total corneal power, corneal thickness maps, higher order aberrations, and densitometry. Statistical analysis will be similar to that describe above, linear mixed effects regression using treatment assignment and baseline values as covariates, using the same template as we did for BSCVA.

### Missing data

For missing outcomes such as visual acuity or scar size, we will use last observation carried forward (LOCF) as well as multiple imputation-based analysis. Additionally, we will conduct sensitivity analyses in which the data are not assumed missing at random, to assess how extreme the missing values would need to be to change the conclusions of the study.

### Adverse events and statistical analyses

All adverse events will be tabulated and reported. Adverse events will regularly be reported to the medical monitor and CCC. Serious adverse events are reported within 24 h to the medical monitor.

#### TPK/perforation

A Cox proportional hazards model will estimate the hazard of perforation, defined as perforation (flat anterior chamber with presence of iris plugging a defect in the cornea or seidel positivity) or the need for TPK while correcting for baseline infiltrate depth.

### Post-trial care

We will inform participants of trials results after full trial completion. For ancillary and post-trial care, we have trial insurance to compensate those who suffer harm from trial participation although this is not anticipated.

### Interim analysis

Interim reports for the DSMC are prepared by the data coordinating center. These reports include (a) recruitment overall and by study site, (b) compliance, and (c) retention. The reports will also list study outcomes, including 6-month BSCVA and microbiological outcomes, and all adverse outcomes, including mortality and perforations. All adverse events are tabulated and reported. Statistical comparisons will be conducted using Fisher’s exact test, but with the caution that failure to find a statistically significant difference cannot be used to infer a lack of risk difference, since the study is not powered to examine rare outcomes. Procedures for reporting both adverse events and serious adverse events, including notification of the medical monitor, were reviewed by the DSMC prior to opening enrollment. We will categorize adverse events, severe adverse events, and events of interest following recommended best practices for clinical trial monitoring and reporting [11].

### Sample size calculation

The trial’s sample size calculation was based on the primary outcome, 6-month BSCVA. We informed the calculation with measurements from the first Steroids for Corneal Ulcers Trial (SCUT), among patients enrolled with between 20/60 and 20/400 vision. The SCUT trial measured BSCVA at baseline, 3 months, and 12 months. We conservatively used the 12-month outcome measure for the calculations since there was no 6-month measurement. The standard deviation of BSCVA at 12 months was 0.293 [32]. Since the primary analysis will adjust for baseline BSCVA, we used an estimate of the residual standard deviation, which is.

$$SD_r=SD1-r_2\quad\quad\sqrt{\mathrm{SDr}=\mathrm{SD}1-\mathrm r2}$$

where *r* is the correlation between the baseline measure and primary endpoint. In SCUT, the correlation between baseline and 12-month BSCVA among patients with between 20/60 and 20/400 vision at enrollment was 0.216. We thus assumed a residual standard deviation of

$$0.2931-0.{216}_2\quad\quad\sqrt{=0.2860.2931-0.262=0.286}$$

Assuming a significance level of 0.05, allowing for approximately 15% loss to follow-up, we estimate that we will have 90% power to detect a 1.1-line difference (logMAR 0.11) between groups with 165 study participants per arm (330 total). For the same sample size and under the same assumptions, the detectable difference at 80% power is 1.0-lines (logMAR 0.10). These calculations were based on the standard power formula for the *T*-test (using an estimated residual standard deviation).

### Dissemination plan

This study will comply with the NIH Data Sharing Policy and Policy on the Dissemination of NIH-Funded Clinical Trial Information and the Clinical Trials Registration and Results Information Submission rule. As such, this trial is registered at ClinicalTrials.gov (NCT05110001), and results from this trial will be submitted and published on ClinicalTrials.gov. In addition, every attempt will be made to publish results in peer-reviewed journals and to present these data at national and international meetings. Consistent with the collaborative nature of the proposed research, the PI anticipates sharing all data generated by the study with collaborators. Analytic datasets that will be developed through the project will comply with the NIH Data Sharing Policy. The analytical datasets from this project will include patient-level data generated from the study visits. Data from the trial will be made available upon reasonable request.

## Discussion

Although bacterial corneal ulcers are more common in the USA, fungal and acanthamoeba keratitis (AK) presents a therapeutic challenge to clinicians because of poor outcomes and few treatment options [33–39]. In the tropics, fungal infection can account for upwards of 50% of corneal ulcers [33, 38, 40]. In the USA, fungal keratitis ranges from 35% of corneal ulcers in South Florida [41] to 4% in temperate climates such as Los Angeles [30]. These infections can occur after trauma, with contact lens wear, or after refractive surgery [42, 43]. An outbreak of *Fusarium* keratitis among contact lens wearers was related to the ReNu Moistureloc™ contact lens solution, which was subsequently removed from the market [44].

The best treatment strategies for fungal keratitis have not been well characterized. Topical natamycin, a polyene, is the only antifungal agent approved by the Food and Drug Administration (FDA) for treatment of fungal keratitis. The Mycotic Ulcer Treatment Trials (MUTT) I and II were two NEI-funded randomized double-masked clinical trials that found topical natamycin to be superior to topical voriconazole and no additional benefit of adjuvant oral voriconazole. Two recent randomized clinical trials also failed to demonstrate a benefit of adjuvant intrastromal voriconazole or adjuvant UVX in the treatment fungal keratitis [18, 45]. However, natamycin is fungistatic and has limited penetration into the corneal layers [46]. Furthermore, outcomes of fungal keratitis with topical natamycin are extremely poor as demonstrated in MUTT II where approximately 50% of patients had full thickness corneal perforation or required TPK despite topical natamycin, topical voriconazole, and adjuvant oral voriconazole.

Although much less common, acanthamoeba keratitis (AK) may have the most prolonged and severe course of any corneal infection. AK is typically related to contact lens use and the incidence of these infections varies from as low as 1% to 4–8% of culture-positive microbial keratitis cases in countries where contact lens use is common [47]. Topical biguanides such as chlorhexidine 0.02% and polyhexamethylene biguanide (PHMB) 0.02% are thought to be the most effective available medical therapy. However, large series suggest that only 60% of patients achieve complete cure with medical therapy alone by 1 year and that almost 50% end up with a poor outcome, defined as requiring TPK or having visual acuity less than 20/80 [48]. Furthermore, these medications are highly toxic and cause permanent damage to delicate ocular structures such as limbal stem cells and trabecular meshwork [49].

Corneal cross-linking (CXL) is a novel prospective therapy that may simultaneously reduce both ocular pathogens and inflammatory cells and strengthen the cornea [1–3]. CXL with riboflavin (UVX) and rose bengal with green light (RB-PDT) are both effective in vitro against common bacterial ocular pathogens, such as *Pseudomonas aeruginosa* and *Streptococcus pneumoniae* [17, 50]. However, UVX appears to have much less effect on fungal and acanthamoeba organisms in vitro and one randomized clinical trial also did not show a benefit of adjuvant UVX in the treatment of filamentous fungal keratitis patients [18].

In vitro RB-PDT appears to be much more effective against fungal and acanthamoeba isolates [22, 23]. Rose bengal (RB) is one of the most commonly used dyes in the diagnosis of ocular surface disease [51]. Rose bengal is an effective photosensitizer, readily converting triplet oxygen (^3^O_2_) to produce high singlet oxygen (^1^O_2_) yields with exposure to green light [52]. Although RB dye penetration is to approximately 100 μm into the stroma, subsequent free radical formation occurs up to 1/3 of the corneal stromal depth [26, 53]. The ability of RB to continue free radical formation is self-limited after photo-irradiation has ceased [54]. Multiple in vitro and ex vivo studies have suggested that RGX may be safer than UVX. Wound healing studies found more corneal haze and slower wound healing after UVX compared with RGX [9]. Rabbit studies have demonstrated the safety of RGX on limbal stem cells and endothelium and found anterior stromal keratocyte damage in RGX comparable to epithelial debridement alone [13, 14]. By contrast, UVX causes an immediate decrease in the sub-epithelial nerve plexus and loss of keratocytes in the anterior one third of the corneal stroma, although this recovers after a few months [55, 56].

Smear- and culture-negative ulcers represent another therapeutic challenge for clinicians. Up to 60% of corneal cultures are smear and culture negative [57]. When these patients do not improve with topical antibiotics alone, clinicians must decide what alternative medical therapy to introduce. There is little guidance in the literature on how to manage these patients. These cases are challenging to study since they represent different underlying etiologies and one medical therapy is unlikely to address all of them. RB-PDT is unique in its potential to address bacterial, fungal, and parasitic infections making it a particularly attractive novel therapy.

Limitations to our study include the fact that while our study design is practical in terms of resources, the organisms have different clinical courses will likely respond differently to RB-PDT. It is true that these cases are typically not studied together since one medical therapy is unlikely to address all of the underlying etiologies. RB-PDT is unique in its potential to address bacterial, fungal, and parasitic infections, making it possible to enroll all of these ulcers in one study. This also makes RB-PDT a particularly appealing therapy for smear- and culture-negative cases, which are common and are a therapeutic challenge for clinicians [57]. Here, we propose pre-specified subgroup analyses for acanthamoeba, fungal, and smear/culture negative, to analyze the effects of RB-PDT on each type of ulcer.

## Conclusion

Here, we explore a novel adjunctive therapy for the treatment of fungal, acanthamoeba, and smear- and culture-negative keratitis. Reducing the global burden of vision loss from corneal opacification will likely require a multidisciplinary approach including corneal ulcer prevention, novel antimicrobial agents, and adjunctive therapies such as RB-PDT.

## Trial status

Protocol version 3.0 last edited March 15, 2023. Recruitment began in January 2022 and is expected to last until approximately January 2025.

### Supplementary Information

Supplementary Material 1

Supplementary Material 2

## Data Availability

Every attempt will be made to publish results in peer-reviewed journals and to present these data at national and international meetings. Consistent with the collaborative nature of the proposed research, the PI anticipates sharing all data generated by the study with collaborators. Analytic datasets that will be developed through the project will comply with the NIH Data Sharing Policy. The analytical datasets from this project will include patient-level data generated from the study visits. Data from the trial will be made available upon reasonable request.
