# Supplementary material for: A double-masked, sham-controlled trial of rose bengal photodynamic therapy for the treatment of fungal and acanthamoeba keratitis: Rose Bengal Electromagnetic Activation with Green Light for Infection Reduction (REAGIR) study
Source: Trials. 2024 Aug 28;25:566. doi: 10.1186/s13063-024-08376-3 (PMC11351027; doi:10.1186/s13063-024-08376-3)
Supplement: Supplementary file 2 — Supplementary Material 2 [file 13063_2024_8376_MOESM2_ESM.docx]

**Steroids and Cross-linking for Ulcer Treatment (SCUT II)**

and

**Rose Bengal Electromagnetic Activation with Green light for Infection Reduction (REAGIR)**

**Trials**

**Protocol Number: 2.0**

**National Clinical Trial (NCT) Identified Numbers: NCT04097730, NCT05110001**

**Principal Investigators:** **Tom Lietman and Jennifer Rose-Nussbaumer**

**Sponsor: University of California, San Francisco**

**Funded by: National Eye Institute**

**Version Number: v3.0**

**15 March 2023**

**Summary of Changes from Previous Version:**

| **Affected Section(s)** | **Summary of Revisions Made** | **Rationale** |
| --- | --- | --- |
|  | Addition of Federal University of Sao Paulo as REAGIR enrollment center |  |
|  | Correction to concentration of Rose Bengal: 0.1% RB in 0.9% sodium chloride |  |
|  | Clarification: Tumbling E chart only used for patients who don’t read letters |  |

Table of Contents

[STATEMENT OF COMPLIANCE 1](#_Toc129768074)

[1 PROTOCOL SUMMARY 1](#_Toc129768075)

[1.1 Synopsis 1](#_Toc129768076)

[1.2 SchemA 7](#_Toc129768077)

[1.3 Schedule of Activities (SoA) 8](#_Toc129768078)

[2 INTRODUCTION 8](#_Toc129768079)

[2.1 Study Rationale 8](#_Toc129768080)

[2.2 Background 10](#_Toc129768081)

[2.3 Risk/Benefit Assessment 13](#_Toc129768082)

[2.3.1 Known Potential Risks 13](#_Toc129768083)

[2.3.2 Known Potential Benefits 13](#_Toc129768084)

[2.3.3 Assessment of Potential Risks and Benefits 13](#_Toc129768085)

[3 OBJECTIVES AND ENDPOINTS 14](#_Toc129768086)

[4 STUDY DESIGN 16](#_Toc129768087)

[4.1 Overall Design 16](#_Toc129768088)

[4.2 Scientific Rationale for Study Design 17](#_Toc129768089)

[4.3 Justification for Dose 17](#_Toc129768090)

[4.4 End of Study Definition 17](#_Toc129768091)

[5 STUDY POPULATION 17](#_Toc129768092)

[5.1 Inclusion Criteria 17](#_Toc129768093)

[5.2 Exclusion Criteria 18](#_Toc129768094)

[5.3 Lifestyle Considerations 18](#_Toc129768095)

[5.4 Screen Failures 18](#_Toc129768096)

[5.5 Strategies for Recruitment and Retention 18](#_Toc129768097)

[6 STUDY INTERVENTION 19](#_Toc129768098)

[6.1 Study Intervention(s) Administration 19](#_Toc129768099)

[6.1.1 Study Intervention Description 19](#_Toc129768100)

[6.1.2 Dosing and Administration 21](#_Toc129768101)

[6.2 Preparation/Handling/Storage/Accountability 22](#_Toc129768102)

[6.2.1 Acquisition and accountability 22](#_Toc129768103)

[6.2.2 Formulation, Appearance, Packaging, and Labeling 22](#_Toc129768104)

[6.2.3 Product Storage and Stability 22](#_Toc129768105)

[6.2.4 Preparation 23](#_Toc129768106)

[6.3 Measures to Minimize Bias: Randomization and Blinding 23](#_Toc129768107)

[6.4 Study Intervention Compliance 23](#_Toc129768108)

[6.5 Concomitant Therapy 23](#_Toc129768109)

[6.5.1 Rescue Medicine 23](#_Toc129768110)

[7 STUDY INTERVENTION DISCONTINUATION AND PARTICIPANT DISCONTINUATION/WITHDRAWAL 24](#_Toc129768111)

[7.1 Discontinuation of Study Intervention 24](#_Toc129768112)

[7.2 Participant Discontinuation/Withdrawal from the Study 24](#_Toc129768113)

[7.3 Lost to Follow-Up 24](#_Toc129768114)

[8 STUDY ASSESSMENTS AND PROCEDURES 24](#_Toc129768115)

[8.1 Efficacy Assessments 24](#_Toc129768116)

[8.2 Safety and Other Assessments 25](#_Toc129768117)

[8.3 Adverse Events and Serious Adverse Events 25](#_Toc129768118)

[8.3.1 Definition of Adverse Events (AE) 25](#_Toc129768119)

[8.3.2 Definition of Serious Adverse Events (SAE) 25](#_Toc129768120)

[8.3.3 Classification of an Adverse Event 25](#_Toc129768121)

[8.3.4 Time Period and Frequency for Event Assessment and Follow-Up 26](#_Toc129768122)

[8.3.5 Adverse Event Reporting 27](#_Toc129768123)

[8.3.6 Serious Adverse Event Reporting 27](#_Toc129768124)

[8.3.7 Reporting Events to Participants 27](#_Toc129768125)

[8.3.8 Events of Special Interest 27](#_Toc129768126)

[8.3.9 Reporting of Pregnancy 27](#_Toc129768127)

[8.4 Unanticipated Problems 27](#_Toc129768128)

[8.4.1 Definition of Unanticipated Problems (UP) 27](#_Toc129768129)

[8.4.2 Unanticipated Problem Reporting 28](#_Toc129768130)

[8.4.3 Reporting Unanticipated Problems to Participants 28](#_Toc129768131)

[9 STATISTICAL CONSIDERATIONS 29](#_Toc129768132)

[9.1 Statistical Hypotheses 29](#_Toc129768133)

[9.2 Sample Size Determination 30](#_Toc129768134)

[9.3 Populations for Analyses 31](#_Toc129768135)

[9.4 Statistical Analyses 32](#_Toc129768136)

[9.4.1 Analysis of the Primary Efficacy Endpoint 32](#_Toc129768137)

[9.4.2 Analysis of the Secondary Endpoints 32](#_Toc129768138)

[9.4.3 Safety Analyses 33](#_Toc129768139)

[10 SUPPORTING DOCUMENTATION AND OPERATIONAL CONSIDERATIONS 33](#_Toc129768140)

[10.1 Regulatory, Ethical, and Study Oversight Considerations 33](#_Toc129768141)

[10.1.1 Informed Consent Process 33](#_Toc129768142)

[10.1.2 Study Discontinuation and Closure 34](#_Toc129768143)

[10.1.3 Confidentiality and Privacy 34](#_Toc129768144)

[10.1.4 Future Use of Stored Specimens and Data 35](#_Toc129768145)

[10.1.5 Key Roles and Study Governance 36](#_Toc129768146)

[10.1.6 Safety Oversight 36](#_Toc129768147)

[10.1.7 Clinical Monitoring 36](#_Toc129768148)

[10.1.8 Quality Assurance and Quality Control 36](#_Toc129768149)

[10.1.9 Data Handling and Record Keeping 37](#_Toc129768150)

[10.1.10 Protocol Deviations 37](#_Toc129768151)

[10.1.11 Publication and Data Sharing Policy 38](#_Toc129768152)

[10.1.12 Conflict of Interest Policy 39](#_Toc129768153)

[10.2 Additional Considerations 39](#_Toc129768154)

[10.3 Abbreviations 39](#_Toc129768155)

[10.4 Protocol Amendment History 40](#_Toc129768156)

[11 REFERENCES 41](#_Toc129768157)

# STATEMENT OF COMPLIANCE

The trial will be carried out in accordance with International Conference on Harmonisation Good Clinical Practice (ICH GCP) and the following:

- United States (US) Code of Federal Regulations (CFR) applicable to clinical studies (45 CFR Part 46, 21 CFR Part 50, 21 CFR Part 56, 21 CFR Part 312, and/or 21 CFR Part 812)

National Institutes of Health (NIH)-funded investigators and clinical trial site staff who are responsible for the conduct, management, or oversight of NIH-funded clinical trials have completed Human Subjects Protection and ICH GCP Training.

The protocol, informed consent form(s), recruitment materials, and all participant materials will be submitted to the Institutional Review Board (IRB) for review and approval. Approval of both the protocol and the consent form must be obtained before any participant is enrolled. Any amendment to the protocol will require review and approval by the IRB before the changes are implemented to the study. In addition, all changes to the consent form will be IRB-approved; a determination will be made regarding whether a new consent needs to be obtained from participants who provided consent, using a previously approved consent form.

# PROTOCOL SUMMARY

## Synopsis

| **Title:** | Steroids and Cross-linking for Ulcer Treatment (SCUT II) and  Rose Bengal Electromagnetic Activation with Green light for Infection Reduction (REAGIR) | |  |
| --- | --- | --- | --- |
| **Study Description:** | The Steroids and Cross-linking for Ulcer Treatment (SCUT II) and Rose Bengal Electromagnetic Activation with Green light for Infection Reduction (REAGIR) trials are international, randomized, double-masked, sham and placebo-controlled, clinical trials. The purpose of these studies is to determine differences in 6-month visual acuity between medical antimicrobial treatments alone versus antimicrobial treatment plus corneal cross-linking (CXL), as well as to further evaluate findings from subgroup analyses of SCUT.  **SCUT II**  Patients presenting to one of the Aravind Eye Hospitals in India, to the University of California San Francisco (UCSF), or to Bascom Palmer Eye Institute at the University of Miami with smear-positive typical (i.e. non-*Nocardia or Mycobacteria*) bacterial corneal ulcers and moderate to severe vision loss, defined as Snellen visual acuity of 20/40 or worse, will be eligible for inclusion. Those who agree to participate will be randomized to one of three treatment groups:   - Group 1, Standard therapy: topical 0.5% moxifloxacin plus topical placebo plus sham UVX - Group 2, Early steroids: topical 0.5% moxifloxacin plus topical difluprednate 0.05% plus sham UVX - Group 3, UVX plus early steroids: topical 0.5% moxifloxacin plus topical difluprednate 0.05% plus UVX   **REAGIR**  Patients presenting to one of the Aravind Eye Hospitals in India or to the Federal University of São Paulo in Brazil (UNIFESP) with either smear or culture positive fungal or acanthamoeba keratitis or smear and culture negative corneal ulcers and moderate to severe vision loss, defined as Snellen visual acuity of 20/40 or worse, will be eligible for inclusion. Those who agree to participate will be randomized to one of two treatment groups:   - Group 4, Sham RB-PDT: topical chlorhexidine gluconate 0.02%/PHMB 0.02% (acanthamoeba), moxifloxacin 0.5% (smear/culture negative) or natamycin 5%/Amphotericin B 0.15% (fungal keratitis) plus sham RB-PDT - Group 5, RB-PDT: topical chlorhexidine gluconate 0.02%/PHMB 0.02% (acanthamoeba), moxifloxacin 0.5% (smear/culture negative) or natamycin 5%/Amphotericin B 0.15% (fungal keratitis) plus RG-PDT | |  |
| **Objectives:** | **Specific Aim 1: To determine if corneal cross-linking with riboflavin (UVX) is a beneficial adjuvant in the treatment of smear- and/or culture-positive bacterial ulcers.**   - 1. We anticipate that UVX will result in better best spectacle corrected visual acuity (BSCVA) at 6 months compared with antibiotic alone.   2. We anticipate that UVX will result in faster microbiological cure rates, smaller scar size and lower rate of corneal perforation compared with antibiotic alone.   **Specific Aim 2: To determine if early topical steroids are a beneficial adjuvant in the treatment of smear- and/or culture-positive bacterial ulcers.**   1. We hypothesize that those randomized to early topical steroids will have improved BSCVA at 6 months compared with antibiotic alone.   **Specific Aim 3: To determine which ulcer characteristics predict the most benefit from the addition of adjuvant corneal cross-linking and/or early steroids.**   1. We hypothesize that culture positive bacterial ulcers with drug resistant organisms, as measured by MIC_50_, will benefit more from adjuvant UVX than those with antibiotic susceptible organisms. | |  |
|  | **Specific Aim 4:** **To determine if cross linking with rose bengal (RB-PDT) is a beneficial adjuvant in the treatment of fungal, acanthamoeba, and smear or culture negative corneal ulcers.**   1. We hypothesize that there will be improved visual acuity at 6 months among those randomized to adjuvant RB-PDT after controlling for baseline visual acuity. 2. We anticipate that there will be smaller infiltrate/scar size and decreased rate of perforation and/or need for TPK at 6 months among RB-PDT treated patients. | |  |
| **Endpoints:** | Primary Endpoint: Best spectacle-corrected visual acuity at 6 months  Secondary Endpoints:   - Microbiological cure on repeat smear and culture at Day 2 - Interaction of minimum inhibitory concentration (MIC) and CXL on best spectacle-corrected visual acuity - Infiltrate/scar size/depth, as measured by clinical exam, clinical photographs, Pentacam and OCT at week 3 and months 3, 6, and 12 - Adverse events including rate of perforation/need for TPK - BSCVA at Day 1, 3 weeks, 3 months, and 12 months - Astigmatism, higher order aberrations, topography, and densitometry as measured on Pentacam at week 3 and months 3, 6, and 12 - Corneal thickness and scar size/depth as measured by AS-OCT at week 3 and months 3, 6, and 12 - Basal nerve plexus, white blood cell count, and keratocyte density on confocal microscopy at week 3 and months 3, 6, and 12 - Visual function questionnaire (VFQ) will be compared between groups at 6 months, controlling for Day 1 VFQ - Pain scale at baseline, Day 1, and Day 3 - Subgroup analysis of study participants receiving prior topical antimicrobial therapy | |  |
| **Study Population:** | 279 participants with bacterial corneal ulcers (SCUT II) and 330 participants with fungal, acanthamoeba, or smear/culture negative corneal ulcers (REAGIR) will be enrolled at Aravind Eye Hospitals in Coimbatore, Madurai, Pondicherry, and Tirunelveli in Tamil Nadu, South India, at the Federal University of São Paulo, Brazil, at the University of California San Francisco, USA, and at the University of Miami. We anticipate that participants from India will be native to India, an ethnic group often reported as “South Asian”. Previous studies at Aravind suggest that approximately 39-50% of presenting ulcers will be in women. At UCSF, patients will be a mixture of White (non-Hispanic), Hispanic, African-American, and Asian, and will be an estimated 50% female. The original SCUT trial had a median age of 53 years, with interquartile range of 40-61 years, and we expect a similar age range in this study. Participants from Bascom Palmer Eye Institute will be patients that live in South Florida and the Caribbean islands around South Florida. South Florida has a mixture of Hispanic patients, White (non-Hispanic), and African American. Previous studies from Bascom Palmer have had a patient population younger than the SCUT trial. Participants from Brazil will be a mixture of White (non-Hispanic), Hispanic, African American, and Asian, and will be an estimated 50% female. Our most recent epidemiological study of infectious keratitis cases at Hospital Sao Paulo at the Federal University of Sao Paulo showed that the population had a mean age of 48 ± 21 years, and we expect a similar age range in this study. | |  |
| **Phase:** | N/A | |  |
| **Description of Sites/Facilities Enrolling Participants:** | Participants will be enrolled at four sites in India, one site in Brazil, and two sites in the United States. In India, participants will be enrolled at Aravind Eye Hospitals in Tamil Nadu: Coimbatore, Madurai, Pondicherry, and Tirunelveli. Patients will be enrolled at the Cornea Clinic in each hospital. In the United States, participants will be enrolled at the Proctor Foundation clinic at the University of California, San Francisco in San Francisco, CA, and at Bascom Palmer Eye Institute at the University of Miami in Miami, FL. In Brazil, participants will be enrolled at Hospital Sao Paulo, the University hospital of Escola Paulista de Medicina at the Federal University of Sao Paulo (Universidade Federal de Sao Paulo – UNIFESP). | |  |
| **Description of Study Intervention:** | **SCUT II**  Participants with bacterial corneal ulcers will be randomized in a 1:1:1 fashion to one of three arms:   - Group 1, Standard therapy: topical 0.5% moxifloxacin plus topical placebo plus sham UVX - Group 2, Early steroids: topical 0.5% moxifloxacin plus topical difluprednate 0.05% plus sham UVX - Group 3, UVX plus early steroids: topical 0.5% moxifloxacin plus topical difluprednate 0.05% plus UVX   Participants in Groups 1, 2, & 3 will begin study drug (topical difluprednate 0.05% or placebo) within 24 hours after enrollment. Participants in Group 3 will receive UVX (modified Dresden protocol), and Groups 1 & 2 will receive sham UVX. All participants will receive a 30-minute loading dose of topical 0.1% riboflavin and 20% dextran T500 drops every 2 minutes. For Group 3, this will be followed by exposure to UV-A light at a wavelength of 365 nm with an irradiance of 3 mW/cm^2^ for 30 minutes for a total dose of 5.4 J/cm^2^ (UV lamp: PESCHKE Meditrade GmbH, Hueneberg, Switzerland for India; Avedro KXL System, Waltham, MA, USA for USA). During irradiation patients will continue to receive topical riboflavin at 5-minute intervals. For Groups 1 & 2, sham UVX simulates this experience however the light will be shined adjacent to the patient, careful to avoid exposure to the cornea. In place of riboflavin we will use either saline drops or saline drops dyed with fluorescein. All study participants will have repeat corneal cultures 30 minutes after the UVX or sham UVX procedure.  After UVX/sham UVX and a repeat culture, antibiotics will be initiated. All participants will receive 0.5% topical moxifloxacin drops every 1 hour for 2 days, and then every 2 hours while awake until resolution of the epithelial defect.  Participants in Groups 2 & 3 will receive one drop of 0.05% difluprednate four times daily beginning 24 hours after the initiation of antibiotics for 1 week, decreased by 1 drop weekly for a total of 4 weeks of steroid therapy. Participants in Group 1 will receive topical placebo in place of steroids, with the same medication schedule described above.  **REAGIR**  Participants with fungal, acanthamoeba, or smear/culture negative corneal ulcers will be randomized in a 1:1 fashion to one of two arms:   - Group 4, Sham RB-PDT: topical biguanide (acanthamoeba), moxifloxacin 0.5% (smear/culture negative) or polyene (fungal keratitis) plus sham RB-PDT - Group 5, RB-PDT: topical biguanide (acanthamoeba), moxifloxacin 0.5% (smear/culture negative) or polyene (fungal keratitis) plus RG-PDT   Participants in Groups 4 and 5 will begin topical antimicrobial at enrollment. We will use every hour topical biguanide for acanthamoeba, moxifloxacin 0.5% for smear/culture negative, and polyene for fungal keratitis. Topical agents will be applied to the affected eye every hour while awake for the first week, then every 2 hours while awake until 3 weeks after enrollment. Thereafter, application will be tapered at the discretion of the treating physician based on response to therapy.  RB-PDT/sham RB-PDT will occur within 48 hours of randomization. For patients with acanthamoeba keratitis, de-epithelialize central 9mm of the cornea. For Group 5, Rose Bengal (0.1% RB in 0.9% sodium chloride) will be applied in 3-minute intervals to the de-epithelialized cornea for 30 minutes followed by irradiation with a 6mW/cm^2^ custom-made green LED source for 15 minutes (5.4J/cm^2^). They will undergo repeat cornea cultures within 24 hours after the procedure. Participants in Group 4 will undergo a sham procedure using topical balanced salt solution and pen light covered with a green filter. | |  |
|  | |  |  |
| **Study Duration:** | | 5 years | |
| **Participant Duration:** | | 12 months | |

## SchemA

Prior to

Screen potential participants by inclusion and exclusion criteria; obtain history, document. Screening assessments include:

pinhole visual acuity, culture/smear, AS-OCT, and confocal microscopy

Enrollment

Obtain informed consent

Randomize

Group 5:

RB-PDT

N= 165

Group 4:

Sham RB-PDT

N= 165

Perform additional baseline assessments:

Baseline form, slit lamp examination, Pentacam, clinical photography, IOP, pain scale

Day 0

Slit lamp examination, begin study drug, BSCVA, IOP, VFQ, pain scale

Day 1

CXL or sham CXL, repeat scraping

Day 2

Pain scale

Day 3

Follow-up assessments of study endpoints and safety:

Follow-up form, IOP, AS-OCT, confocal microscopy, Pentacam, clinical photography, slit lamp examination, BSCVA

Week 3

Follow-up assessments of study endpoints and safety:

Follow-up form, IOP, AS-OCT, confocal microscopy, Pentacam, clinical photography, slit lamp examination, BSCVA

- - 1. Month

Follow-up assessments of study endpoints and safety:

VFQ, Follow-up form, IOP, AS-OCT, confocal microscopy, Pentacam, clinical photography, slit lamp examination, BSCVA

6 Month

Follow-up assessments of study endpoints and safety:

Follow-up form, Final form, IOP, AS-OCT, confocal microscopy, Pentacam, clinical photography, slit lamp examination, BSCVA

- - 1. Month

## Schedule of Activities (SoA)

|  | Visit 1  Day 0 | Visit 2  Day 1 | Visit 3  Day 2 | Visit 4  Day 3 | Visit 5  3-Week  Follow-Up | Visit 6  3-Month Follow-Up | Visit 7  6-Month  Follow-Up | Visit 8  12-Month Follow-Up |
| --- | --- | --- | --- | --- | --- | --- | --- | --- |
| Forms |  |  |  |  |  |  |  |  |
| Consent & Authorization | X |  |  |  |  |  |  |  |
| Baseline Form | X |  |  |  |  |  |  |  |
| Clinical Drawing | X | X |  |  | X | X | X | X |
| VFQ |  | X |  |  |  |  | X |  |
| Follow-up Form |  | X |  |  | X | X | X | X |
| Final Form |  |  |  |  |  |  |  | X |
| Procedures |  |  |  |  |  |  |  |  |
| CXL/Sham CXL |  |  | X |  |  |  |  |  |
| Tests |  |  |  |  |  |  |  |  |
| IOP | X | X |  |  | X | X | X | X |
| Pain Scale | X | X |  | X |  |  |  |  |
| AS-OCT | X |  |  |  | X | X | X | X |
| Confocal Microscopy | X |  |  |  | X | X | X | X |
| Pentacam Topography | X |  |  |  | X | X | X | X |
| Clinical Photography* | X |  |  |  | X | X | X | X |
| Slit Lamp Examination | X | X |  |  | X | X | X | X |
| BSCVA/ETDRS/MRx |  | X |  |  | X | X | X | X |
| Pinhole Visual Acuity | X |  |  |  |  |  |  |  |
| Culture/Smear | X |  | X^+^ |  |  |  |  |  |
| Total Visit Time | 2 hours | 2 hours | 3 hours | 0.5 hours | 1 hour | 1 hour | 1 hour | 1 hour |
| *Clinical photographs also taken upon adverse events | | | | | | | | |
| ^+^Repeat scraping and culture of corneal ulcer done at time of CXL for Participants in Groups 1, 2, and 3; and within 24 hours of CXL for Groups 4 and 5 | | | | | | | | |

# INTRODUCTION

## Study Rationale

**Although antibiotics are successful at achieving microbiological cure in infectious keratitis, outcomes are often poor due to corneal scarring.** Randomized trials comparing different antibiotic treatments have not been able to demonstrate superiority of one antibiotic over another.[^1^](#_ENREF_1) During acute infection pathogens, keratocytes and other inflammatory cells secrete enzymes that promote protein degradation and keratolysis with resultant opacity and irregular astigmatism. Corneal perforation can also result, requiring urgent surgical intervention with therapeutic penetrating keratoplasty (TPK), which has a poor prognosis compared with penetrating keratoplasty (PKP) performed for visual rehabilitation.[^2^](#_ENREF_2)^,^[^3^](#_ENREF_3)^,^[^4^](#_ENREF_4) *Ideal treatment of corneal ulcers would address both the infection and inflammation.*

**The Steroids for Corneal Ulcer Trial (SCUT U10EY015114) Investigated adjuvant topical steroids in addition to antibiotics to reduce the inflammatory response in bacterial ulcers.** The trial failed to find benefit or harm overall; however, pre-specified subgroup analyses suggested that earlier steroid treatment of large, central, non-*Nocardia* ulcers led to better clinical outcomes.[^5^](#_ENREF_5)^,^[^6^](#_ENREF_6) These subgroup analyses have led some to conclude that topical *corticosteroids may be beneficial for specific subgroups of culture positive bacterial ulcers, and that they were most effective when administered early with appropriate antibiotics.*[*^5^*](#_ENREF_5)*^,^*[*^6^*](#_ENREF_6)

**Corneal cross-linking (CXL) is a novel prospective therapy that may simultaneously reduce both inflammatory cells and bacterial pathogens.**[^7-9^](#_ENREF_7) UV-A + riboflavin is effective *in vitro* against common bacterial ocular pathogens, such as *Pseudomonas aeruginosa* and *Streptococcus pneumoniae*.[^10^](#_ENREF_10) Multiple case reports have suggested potential benefits of UVX for treatment of bacterial keratitis, including resolution of resistant infection, halting of progressive melting and symptomatic improvement.[^11-14^](#_ENREF_11) In one small case series bacterial infections resolved even though patients were treated exclusively with photo-chemically activated riboflavin.[^15^](#_ENREF_15) Therapies to reduce inflammation may improve outcomes, however there are concerns about potentiating infection and poor healing. The possibility that UVX may immediately reduce the burden of infectious organisms makes subsequent anti-inflammatory treatment safer.

**Recently another CXL method has been proposed that uses rose bengal (RB) as the photosensitizer and green light (532 nm) and is termed RB-PDT.**[**^16^**](#_ENREF_16) RB-PDT appears to have similar effects on corneal biomechanical properties, is safe for limbal stem cells and endothelium, and demonstrates less toxicity to keratocytes *in vitro* than UVX.[^17-22^](#_ENREF_17) *In vitro* studies have demonstrated limited benefit of UVX for fungal or acanthamoeba keratitis, and one randomized clinical trial also did not show a benefit of adjuvant UVX in filamentous fungal keratitis patients.[^23^](#_ENREF_23) *In vitro* RB-PDT appears to be much more effective against fungal and acanthamoeba isolates.[^24^](#_ENREF_24)^,^[^25^](#_ENREF_25) CXL for infectious keratitis is identified in the literature as Rose Bengal Photodynamic Therapy (RB-PDT) and Photoactivated Chromophore for Infectious Keratitis (PACK-CXL).[^26-28^](#_ENREF_26)

**Cross-linking may reduce the risk of progressive corneal melting and perforation that can complicate the management of corneal ulcers.** Corneal melting occurs in response to proteolytic enzymes released both from pathogens and leukocytes sent to combat infection.[^3^](#_ENREF_3)^,^[^4^](#_ENREF_4) Corneal perforation is a devastating complication, often treated with surgical interventions such as therapeutic penetrating keratoplasty (TPK) which have a poor prognosis compared with penetrating keratoplasty (PKP) performed for visual rehabilitation.[^2^](#_ENREF_2) UVX corneas show increased *in vitro* resistance to keratolysis by collagenase A.[^29^](#_ENREF_29) One non-randomized prospective series of 40 patients found a decreased rate of perforation among those treated with UVX compared with controls despite the fact they had on average larger baseline ulcer size.[^28^](#_ENREF_28) A recent meta-analysis concluded that the probability that UVX was beneficial in inhibiting melting in patients with infectious keratitis was 85% (95% CI 0.77 to 0.91).[^7^](#_ENREF_7) One *ex vivo* study demonstrated that RB-PDT treated corneas have similarly increased resistance to enzymatic degradation by collagenase A compared to UVX despite less penetration of RB into the corneal stroma.[^30^](#_ENREF_30)

**Clinical hypotheses are sometimes best addressed through international research collaborations.** While corneal ulceration is an important cause of disability in technologically advanced countries, it occurs 10-fold more frequently in developing countries.[^8^](#_ENREF_8)^,^[^31^](#_ENREF_31)^,^[^32^](#_ENREF_32) Approximately 2 million people form a corneal ulcer every year in India alone.[^33^](#_ENREF_33)^,^[^34^](#_ENREF_34) The high burden of disease in South Asia permits investigation of the optimal treatment of infectious keratitis to avoid corneal opacification, a leading cause of blindness worldwide.[^35^](#_ENREF_35)^,^[^36^](#_ENREF_36) *Here, we propose an international, 3-arm randomized controlled clinical trial to investigate corneal crosslinking and early topical steroids as adjuvant therapies in the treatment of bacterial corneal ulcers.*

## Background

**Infectious keratitis is a leading cause of monocular blindness worldwide**.[^37^](#_ENREF_37) In the United States bacteria are the most common etiology for corneal ulcers, and they are often associated with contact lens use.[^38^](#_ENREF_38) Despite appropriate antibiotic treatment, severe cases can progress rapidly, and cause permanent vision loss requiring corneal transplantation.[^38^](#_ENREF_38) It has been estimated that the incidence of all forms of infectious keratitis is 28 per 100,000 person-years,[^39^](#_ENREF_39) with bacterial keratitis affecting approximately 30,000 individuals in the United States annually.[^40^](#_ENREF_40) The monocular vision loss associated with corneal ulceration has been shown to reduce vision-related quality of life.[^41^](#_ENREF_41)

**The first step to the treatment of bacterial infection is to achieve microbiological cure**. Clinicians weigh many factors when choosing an antibiotic regimen: broad-spectrum coverage, toxicity, availability and cost, and region -specific epidemiology of pathogens and resistance patterns. We surveyed the Cornea Society listserv regarding empiric antibiotic choice for presumed bacterial ulcers. Despite its toxicity, 55% (*N*=57) of US physicians used fortified topical vancomycin as their first choice due to concerns over the emergence of resistant organisms such as Methicillin-Resistant Staphylococcus Aureus (MRSA).[^42^](#_ENREF_42) However, a recent Cochrane review of high quality, randomized, controlled, clinical trials on the management of bacterial keratitis with topical antibiotics identified no antibiotic strategy that produced a significant difference in the relative risk of treatment success defined as complete re-epithelialization of the cornea or on time to cure.[^1^](#_ENREF_1) *Therefore, we may not be able to dramatically improve clinical outcomes by antibiotic choice alone.*

**The best treatment strategies for fungal keratitis have not been well characterized.** Topical natamycin, a polyene, is the only antifungal agent approved by the Food and Drug Administration (FDA) for treatment of fungal keratitis. The Mycotic Ulcer Treatment Trials (MUTT) I and II, were two NEI-funded randomized double-masked clinical trials that found topical natamycin to be superior to topical voriconazole and no additional benefit of adjuvant oral voriconazole. Two recent randomized clinical trials also failed to demonstrate a benefit of adjuvant intrastromal voriconazole or adjuvant UVX in the treatment of fungal keratitis.[^43^](#_ENREF_43)^,^[^44^](#_ENREF_44) However, natamycin is fungistatic and has limited penetration into the corneal layers.[^45^](#_ENREF_45) Furthermore, outcomes of fungal keratitis with topical natamycin are extremely poor as demonstrated in MUTT II where approximately 50% of patients had full thickness corneal perforation or required TPK despite topical natamycin, topical voriconazole, and adjuvant oral voriconazole.

**Although much less common, acanthamoeba keratitis (AK) may have the most prolonged and severe course of any corneal infection**. AK is typically related to contact lens use and the incidence of these infections varies from as low as 1% to 4-8% of culture positive microbial keratitis cases in countries where contact lens use is common.[^46^](#_ENREF_46) Topical biguanides such as chlorhexidine 0.02% and Polyhexamethylene biguanide (PHMB) 0.02% are thought to be the most effective available medical therapy. However, large series suggest that only 60% of patients achieve complete cure with medical therapy alone by one year and that almost 50% end up with a poor outcome, defined as requiring TPK or having visual acuity less than 20/80.[^47^](#_ENREF_47) Furthermore, these medications are highly toxic and cause permanent damage to delicate ocular structures such as limbal stem cells and trabecular meshwork.[^48^](#_ENREF_48)

**Even if infectious organisms are eliminated, poor vision can result from corneal opacity and irregular astigmatism.** The use of adjuvant corticosteroids has long been debated in the treatment of bacterial keratitis.[^49-51^](#_ENREF_49) Proponents argue that they decrease inflammation and reduce scarring, neovascularization, and stromal melt.[^51-54^](#_ENREF_51) However, others argue that corticosteroids delay epithelial healing and prolong infection.[^55-58^](#_ENREF_55) Three small randomized controlled trials examining the benefit of adjuvant topical steroids for the treatment of corneal ulcers found no difference in visual acuity outcomes or healing times between those randomized to topical antibiotic alone versus topical antibiotic plus topical steroid.[^59-61^](#_ENREF_59)

**The Steroids for Corneal Ulcers Trial (SCUT U10EY015114) is by far the largest randomized controlled trial to have evaluated the role of adjuvant steroids for bacterial ulcers.**[**^62^**](#_ENREF_62) Five hundred study participants with culture-positive bacterial ulcers were enrolled at UCSF, Aravind Eye Hospitals in Madurai, Coimbatore, and Tirunelveli India, and at the Dartmouth-Hitchcock Medical Center in New Hampshire. Patients were randomized to receive either topical prednisolone sodium phosphate 1.0% or topical placebo (sodium chloride 0.9%), started after 48-hours of topical moxifloxacin 0.5%. All patients received 1 drop of their assigned treatment 4 times daily for the first week after enrollment, then 2 times daily for the second week, and then 1 time daily for the third week. After controlling for baseline BSCVA a multiple linear regression showed that corticosteroids provided no significant improvement in 3-month BSCVA over placebo (*P*=0.82). Similarly, there was no difference between arms in secondary outcomes such as rate of re-epithelialization (*P*=0.25), infiltrate/scar size (*P*=0.40) or the number of perforations observed (*P*>0.99). It is also important to note that corticosteroids did not cause an increase in adverse events.[^62^](#_ENREF_62)

**Pre-specified subgroup analyses have suggested that earlier treatment of large, central, non-*Nocardia* ulcers did have improved visual acuity outcomes compared with antibiotic alone** (**Figure 1**)*.*[*^6^*](#_ENREF_6) A 12-month SCUT analysis excluding *Nocardia* ulcers found a 1-line visual acuity benefit among those randomized to topical steroid.[^63^](#_ENREF_63) We also found that those treated with steroid earlier, within 2 to 3 days of antibiotics, had 1-line better visual acuity at 3 months.[^5^](#_ENREF_5) These subgroup analyses have led some to conclude that topical *corticosteroids may be beneficial for specific subgroups of culture positive bacterial ulcers, and that they were most effective when administered early with appropriate antibiotics.*[*^5^*](#_ENREF_5)*^,^*[*^6^*](#_ENREF_6)

**Corneal cross-linking may benefit patients with infectious corneal ulcers through direct anti-microbial and anti-inflammatory effects, as well as increased resistance of corneal tissue to enzymatic degradation.**[^7-9^](#_ENREF_7) Photoactivation of riboflavin with UV light results in release of reactive oxygen species that promote chemical covalent bond formation between adjacent collagen molecules. Reactive oxygen species are also thought to have an antiseptic effect against a broad range of pathogens.[^64^](#_ENREF_64) UVX is currently used as a treatment for corneal ectatic disorders such as keratoconus and post-LASIK ectasia and has been shown to strengthen the cornea and allow it to retain its normal shape.[^65-68^](#_ENREF_65) Immediately after UVX there is a decrease in the sub-epithelial nerve plexus and loss of keratocytes in the anterior one-third of the corneal stroma, although this recovers after a few months.[^69^](#_ENREF_69)^,^[^70^](#_ENREF_70) UVX would presumably destroy inflammatory cells in the anterior stroma by similar mechanisms, although this does not appear to have been studied previously.

**To date, three small prospective clinical trials have been conducted to assess the effect of CXL in the treatment of infectious keratitis.** Bamdad et al randomized 32 patients with moderate bacterial keratitis to receive either UVX plus standard therapy versus standard therapy alone.[^71^](#_ENREF_71) Two weeks after the treatment, those receiving UVX had a lower mean grade of ulcer (0.69 vs 1.70; *P*=0.001), smaller area of epithelial defect (*P*=0.001), and smaller area of infiltrate (*P*<0.001) than those receiving standard therapy alone. Mean treatment duration was also shorter in the UVX group (*P*<0.001). Another trial randomized patients with bacterial, fungal, *Acanthamoeba*, or mixed origin keratitis to UVX versus antimicrobial treatment alone.[^28^](#_ENREF_28) While this trial found no difference between groups, it had multiple issues, including inappropriate randomization, vastly different etiologies of infection, and insufficient power.[^72^](#_ENREF_72) A third, small randomized clinical trial investigating cross-linking as adjuvant therapy for deep fungal ulcers at Aravind Eye Hospital in Madurai, India stopped after only enrolling 13 patients due to concern that UVX could increase the rate of perforation in severe fungal ulcers.[^73^](#_ENREF_73) *Given the limitations of these clinical trials and mixed results, it is not known whether UVX is a beneficial adjuvant therapy for infectious keratitis and a well-designed, larger scale randomized clinical trial is warranted.*

***In vitro* Rose Bengal Photodynamic Therapy (RB-PDT) appears to be much more effective against fungal and acanthamoeba isolates**.[^24^](#_ENREF_24)^,^[^25^](#_ENREF_25) Rose Bengal (RB) is one of the most commonly used dyes in the diagnosis of ocular surface disease.[^74^](#_ENREF_74) Rose Bengal is an effective photosensitizer, readily converting triplet oxygen (^3^O_2_) to produce high singlet oxygen (^1^O_2_) yields with exposure to green light.[^75^](#_ENREF_75) Although RB dye penetration is to approximately 100µm into the stroma, subsequent free radical formation occurs up to 1/3 of the corneal stromal depth.[^76^](#_ENREF_76)^,^[^77^](#_ENREF_77) The ability of RB to continue free radical formation is self-limited after photo-irradiation has ceased.[^78^](#_ENREF_78) Multiple *In vitro* and *ex vivo* studies have suggested that RB-PDT may be safer than UVX. Wound healing studies found more corneal haze and slower wound healing after UVX compared with RB-PDT.[^17^](#_ENREF_17) Rabbit studies have demonstrated the safety of RB-PDT on limbal stem cells and endothelium and found anterior stromal keratocyte damage in RB-PDT comparable to epithelial debridement alone.[^21^](#_ENREF_21)^,^[^22^](#_ENREF_22) By contrast, UVX causes an immediate decrease in the sub-epithelial nerve plexus and loss of keratocytes in the anterior one-third of the corneal stroma, although this recovers after a few months.[^69^](#_ENREF_69)^,^[^70^](#_ENREF_70)

**Smear and culture negative ulcers represent another therapeutic challenge for clinicians.** Up to 60% of corneal cultures are smear and culture negative.[^79^](#_ENREF_79) When these patients do not improve with topical antibiotics alone, clinicians must decide what alternative medical therapy to introduce. There is little guidance in the literature on how to manage these patients. These cases are challenging to study since they represent different underlying aetiologies and one medical therapy is unlikely to address all of them. *RB-PDT is unique in its potential to address bacterial, fungal, and parasitic infections making it a particularly attractive novel therapy.*

**CXL may also reduce the risk of corneal perforation in corneal ulceration.** Corneal melting occurs in response to proteolytic enzymes released both from pathogens and leukocytes sent to combat infection.[^3^](#_ENREF_3)^,^[^4^](#_ENREF_4) Corneal perforation is a devastating complication, often treated with therapeutic penetrating keratoplasty (TPK) which has a poor prognosis compared to non-emergent penetrating keratoplasty (PKP) performed for visual rehabilitation.[^2^](#_ENREF_2) Cross-linked corneas show increased *in vitro* resistance to keratolysis by collagenase A.[^29^](#_ENREF_29) One non-randomized prospective series of 40 patients found a decreased rate of perforation among those treated with UVX compared with controls despite the fact they had on average larger baseline ulcer size.[^28^](#_ENREF_28) A recent meta-analysis concluded that the probability that UVX was beneficial in inhibiting melting in patients with infectious keratitis was 85% (95% CI 0.77 to 0.91).[^7^](#_ENREF_7) *UVX may even provide a new option for treatment of corneal melt in select auto-inflammatory conditions.*

**It will still be important to measure the effect of UVX on clinical outcomes such as visual acuity and scar size.** A number of studies have demonstrated the safety and efficacy of UVX for the treatment of keratoconus with follow up in the 5-10 year range.[^80^](#_ENREF_80) However, the observed corneal flattening associated with improved visual acuity outcomes in keratoconus, could result in unexpected topographic changes in infectious keratitis and it is not known what effect UVX has on corneal scarring in these cases.

## Risk/Benefit Assessment

### Known Potential Risks

The main risk of difluprednate is IOP elevation, which may be associated with optic nerve damage or even permanent vision loss if not monitored. We believe that the possible risk of increased IOP is outweighed by the potential benefits for this treatment and will be monitoring IOP routinely while the patients are inpatient during the first 3 days of treatment. There was no evidence in SCUT that steroids potentiated infection.

The process of corneal cross-linking is painless. Adverse events associated with corneal cross-linking appear to be exceedingly rare. A recent publication of 3-year follow-up of 100 keratoconus eyes, found two adverse events: one instance of corneal infection and one case of corneal edema, which resolved within one week.[^81^](#_ENREF_81) In a series of 16 patients with bacterial ulcers and presenting average visual acuity of approximately 20/100 (including one patient with 20/20 vision) no complications or side effects of treatment were observed.[^15^](#_ENREF_15) Additionally, a meta-analysis of the literature concerning cross-linking for infectious keratitis concluded that the available evidence supports the use of cross-linking for the treatment of infectious keratitis.[^7^](#_ENREF_7) All procedures will be performed by ophthalmologists board certified in the study country who are well versed in all planned treatment procedures.

There may be some discomfort during follow-up testing (BSCVA, IOP, slit lamp, Pentacam, confocal microscopy, and slit lamp imaging of the eye), but this will be kept to a minimum. The participant will be asked to tell the doctor if any of this testing feels painful. There may be a medication reaction such as eye irritation, swelling, pain, redness, or discharge. If this occurs the risks of stopping the medication must be weighed against the severity of infection and other treatment options.

### Known Potential Benefits

Blindness from infectious corneal ulcers is a worldwide public health problem. Although antibiotics are successful at achieving microbiological cure, outcomes remain poor. Acute infection can result in scarring, irregular astigmatism, and corneal perforation requiring surgery. There is reasonable evidence that our interventions (UVX, RB-PDT and early steroid treatment) will be beneficial in the treatment of bacterial keratitis. If our hypotheses are correct, there could potentially be a profound societal benefit from adjuvant steroid, UVX, and RB-PDT.

### Assessment of Potential Risks and Benefits

Knowledge gained from this study may help reduce the burden of corneal blindness in countries around the world. Given the minimal risks to participants in this study, we feel the benefits of the important knowledge we expect to gain from this study outweigh the risks.

# OBJECTIVES AND ENDPOINTS

| OBJECTIVES | ENDPOINTS | JUSTIFICATION FOR ENDPOINTS |
| --- | --- | --- |
| Primary |  |  |
| Specific Aim 1: To determine the effect of UVX on best spectacle-corrected visual acuity | Best spectacle-corrected visual acuity at 6 months | The most clinically important outcome for patients |
| Specific Aim 2: To determine the effect of early topical steroids on best spectacle-corrected visual acuity | Best spectacle-corrected visual acuity at 6 months | The most clinically important outcome for patients |
| Specific Aim 4: To determine the effect of RB-PDT on best spectacle-corrected visual acuity | Best spectacle-corrected visual acuity at 6 months | The most clinically important outcome for patients |
| Secondary |  |  |
| Specific Aim 1: To assess the effect of UVX on microbiological cure | Repeat culture at Day 2 | Culture status is highly related to clinical outcomes. This will allow us to assess for development of drug resistance during treatment. |
| To assess the effect of early topical steroids on microbiological cure | Repeat culture at Day 2 | Culture status is highly related to clinical outcomes and one concern with topical steroids is that it might prevent resolution of infection. |
| Specific Aim 4: To assess the effect of RB-PDT on microbiological cure | Repeat culture at Day 2 | Culture status is highly related to clinical outcomes. This will allow us to assess for development of drug resistance during treatment. |
| Specific Aim 3: To assess the relationship between UVX and drug resistant organisms | Interaction of minimum inhibitory concentration (MIC) and UVX on best spectacle-corrected visual acuity | Drug resistance is a concern, and the Infectious Disease Society of America recommends looking for novel treatments for bacterial infections. UVX does not use antibiotics. |
| Specific Aim 4: To assess the relationship between RB-PDT and drug resistant organisms | Interaction of minimum inhibitory concentration (MIC) and RB-PDT on best spectacle-corrected visual acuity | Culture status is highly related to clinical outcomes. This will allow us to assess whether RB-PDT is more effective when organisms are resistant to topical antimicrobials |
| To determine the effect of CXL and/or early topical steroids on corneal healing | Scar size and infiltrate, % epithelial healing, over the 12-month follow-up period | Corneal healing is the primary goal of treatment |
| To determine the effect of CXL and/or early topical steroids on adverse events | Adverse events including corneal perforation and need for therapeutic penetrating keratoplasty, glaucoma over the 12-month follow-up period | To ensure safety of the intervention |
| To determine the effect of CXL and/or early topical steroids on quality of life | Visual function questionnaire at 6 months as compared to Day 1 questionnaire | Quality of life is an important patient outcome and will help determine if any differences in BSCVA are clinically relevant |
| Subgroup analysis of study participants receiving prior topical antimicrobial therapy | MIC, Visual acuity, scar size, % epithelial healing, corneal perforation/TPK | We will evaluate whether antimicrobial resistance as measured by MIC leads to worse visual acuity, scar size, epithelial healing, and higher risk of complications such as TPK. |
| Tertiary/Exploratory |  |  |
| To evaluate the effect of corneal infections and study interventions at the cellular level on the cornea using confocal microscopy | Automated corneal nerve fiber analysis and manual quantitative analysis of epithelial cells, inflammatory cells, and keratocytes, fungal hyphae or acanthameoba cells from confocal microscopy images at baseline, 3 weeks, 3 months, 6 months, and 12 months | This objective will help us to learn more about the pathophysiology of corneal infections as well as the effects of study interventions on keratocytes and white blood cell trafficking; to identify findings on confocal which would guide clinicians regarding the best management of bacterial keratitis; and to validate these findings as potential outcomes for future clinical trials. |
| To assess the effect of study interventions on corneal thinning, scarring, and irregularity using Pentacam Scheimpflug topography | Steep and flat keratometry readings, total astigmatism in diopters, root mean square Zernike polynomials in the central 4mm of the pupil, and densitometry in gray scale units, at baseline, 3 weeks, 3 months, 6 months, and 12 months | While CXL might kill bacteria, there are unknown effects on astigmatism. Steroids may decrease the amount of astigmatism. |
| To assess the effect of study interventions on corneal thinning and scarring using high resolution anterior segment OCT | Central corneal thickness, point of maximal thinning, infiltrate/scar size, and infiltrate/scar depth at baseline, 3 weeks, 3 months, 6 months, and 12 months | We can look for baseline ulcer characteristics which predict response to a particular intervention, for example deep infiltrates may not benefit from CXL |

# STUDY DESIGN

## Overall Design

The Steroids and Cross-linking for Ulcer Treatment (SCUT II) and Rose Bengal Electromagnetic Activation with Green light for Infection Reduction (REAGIR) trials are international, randomized, double-masked, sham and placebo controlled, clinical trials. The purpose of these studies is to determine differences in 6-month visual acuity between medical antimicrobial treatments alone versus antimicrobial treatment plus corneal cross-linking (CXL), as well as to further evaluate findings from subgroup analyses of SCUT.

**SCUT II**

Patients presenting to one of the Aravind Eye Hospitals in India to the University of California San Francisco (UCSF), or to Bascom Palmer Eye Institute at the University of Miami with smear-positive typical (i.e. non-Nocardia or Mycobacteria) bacterial corneal ulcers and moderate to severe vision loss, defined as Snellen visual acuity of 20/40 or worse, will be eligible for inclusion. Those who agree to participate will be randomized to one of three treatment groups:

Group 1, Standard therapy: topical 0.5% moxifloxacin plus topical placebo plus sham UVX

Group 2, Early steroids: topical 0.5% moxifloxacin plus topical difluprednate 0.05% plus sham UVX

Group 3, UVX plus early steroids: topical 0.5% moxifloxacin plus topical difluprednate 0.05% plus UVX

We hypothesize that both UVX and early topical steroids will result in better best spectacle-correct visual acuity at 6 months compared with antibiotic alone. The patient, physicians, microbiologist and refractionist performing the best spectacle-corrected visual acuity (BSCVA) assessment will be masked to the treatment group. Due to the nature of the intervention, the surgeon and technician performing cross-linking will not be masked. All study medications and placebo will be labelled identically to ensure adequate masking of study physicians and patients. An interim analysis will be performed once primary outcome data is available for one third of the patients (see SCUT II **Statistical Analysis Plan (SAP)–- Section 1.5.6** for details).

**REAGIR**

Patients presenting to one of the Aravind Eye Hospitals in India, or to the Federal University of São Paulo in Brazil (UNIFESP) with either smear or culture positive fungal or acanthamoeba keratitis or smear and culture negative corneal ulcers and moderate to severe vision loss, defined as Snellen visual acuity of 20/40 or worse, will be eligible for inclusion. Those who agree to participate will be randomized to one of two treatment groups:

Group 4, (Sham RB-PDT): chlorhexidine gluconate 0.02% (acanthamoeba), moxifloxacin 0.5% (smear/culture negative) or natamycin 5% (fungal keratitis) plus sham RB-PDT

Group 5, RB-PDT: chlorhexidine gluconate 0.02% (acanthamoeba), moxifloxacin 0.5% (smear/culture negative) or natamycin 5% (fungal keratitis) plus RB-PDT

We hypothesize that study participants randomized to RB-PDT will have improved BSCVA, achieve microbiological cure more quickly, have smaller scar sizes and lower perforation rates compared with those who receive topical antibiotic alone. The patient, physicians, microbiologist and refractionist performing the best spectacle-corrected visual acuity (BSCVA) assessment will be masked to the treatment group. Due to the nature of the intervention, the surgeon and technician performing cross-linking will not be masked. As in the SCUT II trial, an interim analysis will be performed once primary outcome data is available for one third of the patients (see REAGIR **Statistical Analysis Plan (SAP)–- Section 3.5** for details).

## Scientific Rationale for Study Design

Randomized control trials are known to be the least biased form of evidence; we have chosen to do a randomized control trial with placebo and sham controls to introduce as little bias as possible. Sham cross-linking and placebo with study drug vehicle are included to maintain masking of the study participant and treating physician. No group is receiving less than the standard of care, which is topical antibiotic or antimicrobial treatment.

## Justification for Dose

Route of administration and dosage of study medications are consistent with standard practice. Topical placebo will be administered in the same way as topical difluprednate to mask the patient, physician, and study staff. The topical placebo solution will have the same clear appearance as difluprednate to maintain masking.

## End of Study Definition

A participant is considered to have completed the study if he or she has completed all phases of the study including the final 12-month visit, as shown in the Schedule of Activities (SoA), **Section 1.3**.

# STUDY POPULATION

## Inclusion Criteria

In order to be eligible to participate, an individual must meet all of the following criteria:

**SCUT II:**

- Corneal ulcer that is smear positive and/or culture positive (within 24 hours) for typical bacteria (i.e. non-*Nocardia* or Myobacteria)
- Moderate to severe vision loss, defined as Snellen visual acuity of 20/40 (6/12) or worse
- Corneal thickness ≥350 µm, as measured on AS-OCT
- Age over 18 years
- Basic understanding of the study as determined by the physician
- Commitment to return for follow up visits

**REAGIR:**

- Presence of smear or culture positive fungal or acanthamoeba ulcer; smear or culture negative ulcer; or any atypical bacteria (such as *Nocardia*)
- Moderate to severe vision loss, defined as Snellen visual acuity of 20/40 (6/12) or worse
- Corneal thickness ≥350 µm, as measured on AS-OCT
- Age over 18 years
- Basic understanding of the study as determined by the physician
- Commitment to return for follow up visits

## Exclusion Criteria

An individual who meets any of the following criteria will be excluded from participation in this study:

- Evidence of concomitant infection on exam, gram stain, or confocal microscopy (i.e. herpes, both bacteria and acanthamoeba on gram stain)
- Impending or frank perforation at recruitment
- Involvement of sclera at presentation
- Presence of desmetocele at recruitment
- Non-infectious or autoimmune keratitis
- History of corneal transplantation
- History of intraocular surgery within the last three months*
- Pinhole visual acuity worse than 20/200 in the unaffected eye
- Participants who are decisionally and/or cognitively impaired

*Recent intraocular surgery is an exclusion to avoid ulcers related to the surgery itself (e.g.: wound infection). Three months post-surgery is generally acceptable, although the ophthalmologist can judge whether, in their opinion, the ulcer is related to the surgery. For example, one month after an uncomplicated cataract surgery might be considered acceptable.

## Lifestyle Considerations

Patients at some Aravind Eye Hospital enrollment centers will be admitted to the inpatient setting for the first 3 days of the study.

## Screen Failures

Screen failures are defined as participants who consent to participate in the clinical trial but are not subsequently randomly assigned to the study intervention or entered in the study. A minimal set of screen failure information is required to ensure transparent reporting of screen failure participants, to meet the Consolidated Standards of Reporting Trials (CONSORT) publishing requirements and to respond to queries from regulatory authorities. Minimal information includes demography, screen failure details, eligibility criteria, and any serious adverse event (SAE). In this study, all patients who are enrolled will be randomized and included in the primary analysis, regardless of whether or not they actually receive the assigned intervention.

## Strategies for Recruitment and Retention

Patients presenting to recruitment sites with smear-positive typical bacterial corneal ulcers, smear or culture positive fungal or acanthamoeba ulcers, or smear or culture negative ulcers with moderate to severe vision loss will be approached for possible inclusion in the study. For eligible patients, the study will be explained in the local language (Tamil at the Aravind Eye Clinics, English or Spanish at UCSF and University of Miami, and Portuguese at UNIFESP) in addition to the risks and benefits of participating in the study. Patients at some Aravind sites will be admitted to the hospital for the first 3 days of the study, ensuring minimal loss to follow-up through day 3. Patients will schedule their follow up visits with the study coordinator while they are in inpatient care. The study coordinator will give the patient written documentation of their upcoming visits, and will follow-up with a phone call as their appointments approach. Previous studies with Aravind and UCSF, including the original Steroids for Corneal Ulcers Trial, the Mycotic Ulcer Treatment Trials I & II, and the small cross-linking for bacterial keratitis feasibility assessment study we performed, have had high retention and leave us confident that this study will have high retention as well.

# STUDY INTERVENTION

## Study Intervention(s) Administration

### Study Intervention Description

**SCUT II:** *Corneal Cross-Linking with Riboflavin*

Participants in Groups 1, 2, & 3 will begin study drug (topical difluprednate 0.05% or placebo) within 24 hours after enrollment.

Participants in Group 3 will receive UVX (modified Dresden protocol), and Groups 1 & 2 will receive sham UVX within 48 hours of enrollment. All participants will receive a 30-minute loading dose of topical 0.1% riboflavin and 20% dextran T500 drops every 2 minutes. For Group 3, this will be followed by exposure to UV-A light at a wavelength of 365 nm with an irradiance of 3 mW/cm^2^ for 30 minutes for a total dose of 5.4 J/cm^2^ (UV lamp: PESCHKE Meditrade GmbH, Hueneberg, Switzerland for India; Avedro KXL System, Waltham, MA, USA for USA). During irradiation patients will continue to receive topical riboflavin at 5-minute intervals. For Groups 1 & 2, sham UVX simulates this experience however the light will be shined adjacent to the patient, careful to avoid exposure to the cornea. In place of riboflavin we will use either saline drops or saline drops dyed with fluorescein.

Moxifloxacin 0.5%

Topical moxifloxacin 0.5% is a fluoroquinolone antibiotic that is used to treat bacterial infections. This is standard therapy for bacterial keratitis. Immediately after UVX/sham UVX and repeat culture, all participants will receive topical moxifloxacin drops every 1 hour for 2 days, and then every 2 hours while awake until resolution of the epithelial defect.

Difluprednate 0.05%

Difluprednate 0.05% is a corticosteroid used to reduce inflammation in the eye. Participants in Groups 2 & 3 will receive one drop of 0.05% difluprednate four times daily beginning 24 hours after the initiation of antibiotics for 1 week, decreased by 1 drop weekly for a total of 4 weeks of steroid therapy.

Placebo

Participants randomized to Group 1 will receive topical placebo in place of topical difluprednate. The placebo will be the vehicle used in difluprednate. Group 1 will receive the topical placebo on the same medication schedule described above for difluprednate.

**REAGIR:** *Corneal Cross-Linking with Rose Bengal*

Participants in Groups 4 and 5 will begin topical antimicrobial at enrollment.

Participants in Group 5 will receive RB-PDT and Group 4 will receive sham RB-PDT within 48 hours of randomization. All participants will receive a 30-minute loading dose of topical Rose Bengal (0.1% RB in 0.9% sodium chloride) which will be applied in 3-minute intervals to the de-epithelialized cornea. This will be followed by irradiation with a 6mW/cm^2^ custom-made green LED source for 15 minutes (5.4J/cm^2^). Participants in Group 4 will undergo a sham procedure using topical balanced salt solution and pen light covered with a green filter. Participants in both groups will undergo repeat cornea cultures within 24 hours after the procedure.

*Rose Bengal 0.1%*

Rose Bengal (RB) is one of the most commonly used dyes in the diagnosis of ocular surface disease.[^74^](#_ENREF_74) Rose Bengal is an effective photosensitizer, readily converting triplet oxygen (^3^O_2_) to produce high singlet oxygen (^1^O_2_) yields with exposure to green light.[^75^](#_ENREF_75) Although RB dye penetration is to approximately 100µm into the stroma, subsequent free radical formation occurs up to 1/3 of the corneal stromal depth.[^76^](#_ENREF_76)^,^[^77^](#_ENREF_77) The ability of RB to continue free radical formation is self-limited after photo-irradiation has ceased.[^78^](#_ENREF_78)

Chlorhexidine gluconate 0.02% or polyhexamethylene biguanide 0.02%

Topical biguanides such as chlorhexidine gluconate 0.02% or PHMB 0.02% are thought to be the most effective available medical therapy for acanthamoeba. Participants in Groups 4 and 5 with acanthamoeba ulcers will receive topical chlorhexidine drops every 1 hour for 2 days, and then every 2 hours while awake, tapering at the clinician’s discretion.

Moxifloxacin 0.5%

At enrollment, participants with smear/culture negative ulcers will receive topical moxifloxacin drops every 1 hour for 2 days, and then every 2 hours while awake, tapering at the clinician’s discretion.

*Natamycin 5%*

Topical natamycin, a polyene, is the only antifungal agent approved by the Food and Drug Administration (FDA) for treatment of fungal keratitis. Participants in Groups 4 and 5 with fungal ulcers will receive topical natamycin every 1 hour for 2 days, and then every 2 hours while awake, tapering at the clinician’s discretion.

Amphotericin B 0.15%

In Brazil, participants in Groups 4 and 5 with fungal ulcers will receive topical amphotericin B 0.15% every 1 hour for 2 days, then every 2 hours while awake, tapering at the clinician’s discretion.

### Dosing and Administration

The schedule of medications for the studies are below:

**SCUT II:** *Dosing schedule for medications*

|  | Group 1:  Standard Therapy | | Group 2:  Early Steroids | | Group 3:  UVX | | |
| --- | --- | --- | --- | --- | --- | --- | --- |
|  | **Dose** | **Duration** | **Dose** | **Duration** | **Dose** | **Duration** | |
| Moxifloxacin 0.5% | Q 1 hours x 2 days, then Q 2 hours while awake | Until resolution of epithelial defect | Q 1 hours x 2 days, then Q 2 hours while awake | Until resolution of epithelial defect | Q 1 hours x 2 days, then Q 2 hours while awake | Until resolution of epithelial defect | |
| Difluprednate 0.05%* |  |  | QID | Starting at 24 hours after initiation of antibiotics for 1 week, then decreasing by 1 drop weekly for a total of 4 weeks of steroid therapy | QID | Starting at 24 hours after initiation of antibiotics for 1 week, then decreasing by 1 drop weekly for a total of 4 weeks of steroid therapy | |
| Riboflavin 0.1% |  |  |  |  | Q2 min | | 60 minutes |
| Homatropine | TID | 3 days | TID | 3 days | TID | | 3 days |

*for patients randomized to Groups 2 & 3 only

Participants randomized to receive topical placebo instead of topical steroids will receive the placebo on the same medication schedule as difluprednate outlined above. If at any time the masked treating physician deems it appropriate to change the patient’s treatment, he/she may do so.

**REAGIR:** *Dosing schedule for medications*

|  | Group 4:  Sham RB-PDT | | Group 5:  RB-PDT | | |
| --- | --- | --- | --- | --- | --- |
|  | **Dose** | **Duration** | **Dose** | **Duration** | |
| Biguanide  (Chlorhexidine gluconate 0.02%  or  PHMB 0.02%) | Q 1 hours x 2 days, then Q 2 hours while awake | Tapered at clinician’s discretion | Q 1 hours x 2 days, then Q 2 hours while awake | Tapered at clinician’s discretion | |
| Moxifloxacin 0.5% | Q 1 hours x 2 days, then Q 2 hours while awake | Tapered at clinician’s discretion | Q 1 hours x 2 days, then Q 2 hours while awake | Tapered at clinician’s discretion | |
| Polyene  (Natamycin 5% or Amphotericin B 0.15%) | Q 1 hours x 2 days, then Q 2 hours while awake | Tapered at clinician’s discretion | Q 1 hours x 2 days, then Q 2 hours while awake | | Tapered at clinician’s discretion |
| Rose Bengal  0.1% RB in 0.9% sodium chloride |  |  | Q 3 min | | 45 minutes |
| Homatropine | TID | 3 days | TID | | 3 days |

## Preparation/Handling/Storage/Accountability

### Acquisition and accountability

Medications and masked placebos will be purchased and distributed in the country of enrollment. At Aravind Eye Hospitals, study medications will be compounded and distributed by Aurolab.

For UCSF and University of Miami, Rancho Park Compounding Pharmacy in Los Angeles, CA will assist with compounding and distributing study medications. Information on current medications and drug allergies will be collected in REDCap and reviewed by Rancho Park before study medication is dispensed.

### Formulation, Appearance, Packaging, and Labeling

Transfer of topical study medication or vehicle (placebo) to an identical bottle by the compounding pharmacy will ensure patient and physician remain masked to treatment arm.

### Product Storage and Stability

All study medications should be stored at 15-25°C and should be protected from sunlight. Expiration dates will be clearly labeled.

### Preparation

All preparation will be performed by the compounding pharmacy and delivered directly to the patient. To that end, patient mailing addresses will be provided to Rancho Park.

## Measures to Minimize Bias: Randomization and Blinding

The DCC at UCSF will create the REDCap database to house the randomization list and store data. Once a patient has been identified for inclusion in the study, the study coordinator will review the inclusion/exclusion criteria checklist, and then input the study eye into the REDCap database.

Patients with bacterial ulcers are eligible for randomization to Groups 1, 2, and 3, while patients with fungal, acanthamoeba, and smear/culture negative ulcers are eligible for randomization to Group 4 or 5. Each study eye will be randomly assigned to a treatment group by REDCap. Randomization will be stratified by site. Stratified, block randomization will ensure that an approximately equal number of patients are randomized to each treatment group by site. In SCUT II (groups 1, 2, 3) exact block size will be randomly permuted (block size of 3 with probability 0.7 and 6 with probability 0.3) and will only be known to the biostatistician. In REAGIR (Groups 4, 5) exact block size will be randomly permuted (block size of 4 with probability 0.7 and 6 with probability 0.3) and will only be known to the biostatistician. Block randomization will be performed using a computer program (Statistical package R; R Foundation for Statistical Computing, Vienna, Austria) by the coordinating site.

Once an eye is enrolled (and only one eye can be enrolled) in the study, the study coordinator will assign the study participant’s eye an ID (alpha-numeric code) and organize the procedure in the operating room within 48 hours of enrollment. Once the study participant has been assigned a study participant ID and randomized to treatment group, they will be included in the intent to treat analysis.

The patient, physician performing repeat scraping and clinical follow up, microbiologist and refractionist performing the BSCVA will be masked to treatment arm. Due to the nature of the surgical intervention, the surgeon and technician performing cross-linking will not be masked. All study medications and placebo will be labeled identically to ensure adequate masking of study physicians and patients.

## Study Intervention Compliance

We will have several measures in place to assess compliance with medications. All Aravind patients will be hospitalized for the first three days of the study, and during this time there will be direct observation of medication administration by study staff. At follow-up visits, patients will be asked to bring their medication bottles to assess compliance. We will also have inpatient and outpatient medication logs to track compliance and missed doses, to be filled out by study staff and the patient, respectively.

## Concomitant Therapy

The masked treating physician will be allowed to change antibiotics or antimicrobials based on sensitivity data from culture results and clinical response to treatment. These changes will be recorded and reported on the follow up form.

### Rescue Medicine

The masked treating physician will be allowed to add or change any therapy deemed necessary, including surgery.

# STUDY INTERVENTION DISCONTINUATION AND PARTICIPANT DISCONTINUATION/WITHDRAWAL

## Discontinuation of Study Intervention

Discontinuation from assigned study intervention does not mean discontinuation from the study, and remaining study procedures should be completed as indicated by the study protocol. If a clinically significant finding is identified (including, but not limited to changes from baseline) after enrollment, the investigator or qualified designee will determine if any change in participant management is needed. Any new clinically relevant finding will be reported as an adverse event (AE).

The data to be collected at the time of study intervention discontinuation will include the following:

- Reason for study intervention discontinuation
- New prescribed treatment

## Participant Discontinuation/Withdrawal from the Study

Participants are free to withdraw from participation in the study at any time upon request. The reason for participant discontinuation or withdrawal from the study will be recorded on a dropout form. Subjects who sign the informed consent form and are randomized but do not receive the study intervention may not be replaced and will be included in the intent to treat analysis. Subjects who sign the informed consent form, and are randomized and receive the study intervention, and subsequently withdraw, or are withdrawn or discontinued from the study, will not be replaced and will be included in the primary analysis.

## Lost to Follow-Up

A participant will be considered lost to follow-up if he or she fails to return for scheduled visits and is unable to be contacted by the study site staff.

The following actions must be taken if a participant fails to return to the clinic for a required study visit:

- The site will attempt to contact the participant and reschedule the missed visit and counsel the participant on the importance of maintaining the assigned visit schedule and ascertain if the participant wishes to and/or should continue in the study.
- Before a participant is deemed lost to follow-up, the investigator or designee will make every effort to regain contact with the participant (where possible, 3 telephone calls and, if necessary, a certified letter to the participant’s last known mailing address or local equivalent methods). These contact attempts should be documented in the participant’s medical record or study file.
- Should the participant continue to be unreachable, he or she will be considered to have withdrawn from the study with a primary reason of lost to follow-up.

# STUDY ASSESSMENTS AND PROCEDURES

## Efficacy Assessments

The following masked efficacy assessments will be performed:

- **Slit lamp examination.** Performed by the treating physician.
- **Visual acuity assessment.** Both pinhole visual acuity and BSCVA/ETDRS/Mrx will be used depending on the time point. Visual acuity will be assessed only by a trained, masked optometrist to ensure proper procedures are followed across sites. See **MOP Section 4.4** for the visual acuity protocol.
- **Imaging assessments**. Non-contact imaging will be performed with HR AS-OCT, Pentacam topography, and slit lamp photography, and contact imaging with confocal microscopy. See **MOP Section 4.6** for detailed descriptions of imaging procedures. Performed by trained study staff.
- **Corneal Gram Stain and Culture***.* Performed by the treating physician. See **MOP Section 4.2** for detailed description of corneal scraping procedures.
- **Visual function questionnaire (VFQ).** The National Eye Institute Visual Function Questionnaire (NEI-VFQ) and the Indian Visual Function Questionnaire (IND-VFQ) will be administered at UCSF/UM and Aravind, respectively. Both are validated questionnaires to evaluate the effect of vision on the patient’s quality of life. Performed by trained study staff.

Please see the SoA in **Section 1.3** of this protocol for a detailed schedule of all study activities.

All of the above assessments, with the exception of the VFQ, will be performed during the screening process. These are standard of care procedures that the patient would receive even if they were not being screened for the study.

The masked treating physician may use the results of these assessments to change the participant’s treatment in any way they deem necessary.

## Safety and Other Assessments

The following assessments will be administered to monitor patient safety:

- **Interval history from the patient** to ask about side effects, etc.
- **Visual acuity assessment.**
- **Intraocular pressure.**
- **Slit lamp exam to identify complications.** Ongoing AEs and SAEs will be followed until resolved.
- **Assessment of study intervention adherenc**e. See Study Intervention Compliance, **Section 6.4**.

## Adverse Events and Serious Adverse Events

### Definition of Adverse Events (AE)

Adverse event means any untoward medical occurrence associated with the use of an intervention in humans, whether or not considered intervention-related (21 CFR 312.32 (a)).

### Definition of Serious Adverse Events (SAE)

An adverse event (AE) or suspected adverse reaction is considered "serious" if, in the view of either the investigator or sponsor, it results in any of the following outcomes: death, a life-threatening adverse event, inpatient hospitalization or prolongation of existing hospitalization, a persistent or significant incapacity or substantial disruption of the ability to conduct normal life functions, or a congenital anomaly/birth defect. Important medical events that may not result in death, be life-threatening, or require hospitalization may be considered serious when, based upon appropriate medical judgment, they may jeopardize the participant and may require medical or surgical intervention to prevent one of the outcomes listed in this definition.

### Classification of an Adverse Event

#### Severity of Event

The following guidelines will be used to describe AE severity.

- **Mild** – Events require minimal or no treatment and do not interfere with the participant’s daily activities.
- **Moderate** – Events result in a low level of inconvenience or concern with the therapeutic measures. Moderate events may cause some interference with functioning.
- **Severe** – Events interrupt a participant’s usual daily activity and may require systemic drug therapy or other treatment. Severe events are usually potentially life-threatening or incapacitating. Of note, the term “severe” does not necessarily equate to “serious”.

#### Relationship to Study INTERVENTION

All adverse events (AEs) – ocular and non-ocular -must have their relationship to study intervention assessed by the clinician who examines and evaluates the participant based on temporal relationship and his/her clinical judgment. The degree of certainty about causality will be graded using the categories below. In a clinical trial, the study product must always be suspect.

- **Related** – The AE is known to occur with the study intervention, there is a reasonable possibility that the study intervention caused the AE, or there is a temporal relationship between the study intervention and event. Reasonable possibility means that there is evidence to suggest a causal relationship between the study intervention and the AE.
- **Not Related** – There is not a reasonable possibility that the administration of the study intervention caused the event, there is no temporal relationship between the study intervention and event onset, or an alternate etiology has been established.

#### Expectedness

The treating physician will be responsible for determining whether an adverse event (AE) is expected or unexpected. An AE will be considered unexpected if the nature, severity, or frequency of the event is not consistent with the risk information previously described for the study intervention.

### Time Period and Frequency for Event Assessment and Follow-Up

The occurrence of an adverse event (AE) or serious adverse event (SAE) may come to the attention of study personnel during study visits and interviews of a study participant presenting for medical care, or upon review by a study monitor.

All AEs including local and systemic reactions not meeting the criteria for SAEs will be captured on the adverse event form. Information to be collected includes event description, time of onset, clinician’s assessment of severity, relationship to study product (assessed only by those with the training and authority to make a diagnosis), and time of resolution/stabilization of the event. All AEs occurring while on study must be documented appropriately regardless of relationship. All AEs will be followed to adequate resolution.

Any medical condition that is present at the time that the participant is screened will be considered as baseline and not reported as an AE. However, if the study participant’s condition deteriorates at any time during the study, it will be recorded as an AE.

Changes in the severity of an AE will be documented to allow an assessment of the duration of the event at each level of severity to be performed. AEs characterized as intermittent require documentation of onset and duration of each episode.

The masked treating physician will record all reportable events with start dates occurring any time after informed consent is obtained until 7 (for non-serious AEs) or 30 days (for SAEs) after the last day of study participation. At each study visit, the investigator will inquire about the occurrence of AE/SAEs since the last visit. Events will be followed for outcome information until resolution or stabilization.

### Adverse Event Reporting

All AEs will be recorded in both the follow-up form and in a specific adverse event form. The SCUT II/REAGIR Data and Safety Monitoring Committee will review Adverse Events at twice yearly committee meetings.

### Serious Adverse Event Reporting

All AEs should be followed until they are resolved or until a stable clinical endpoint is reached. Each AE is to be classified by the Investigator as SERIOUS (SAE) or NONSERIOUS (NSAE). Examples of ocular SAE: TPK, corneal perforation, endophthalmitis, IOP>30. Examples of non-ocular SAE: death, hospitalization, major medical event (eg. MI, stroke).

If an Adverse Event occurs, the investigator must submit the adverse event form in REDCap within 24 hours of the occurrence of the Adverse Event. The investigator must provide written follow-up reports until the SAE or clinically significant AE has resolved or until a stable clinical endpoint is reached. The medical monitor, Stephen McLeod (UCSF), will automatically be notified by the REDCap system when an Adverse Event or Severe Adverse Event is submitted. Because TPKs and corneal perforations are expected outcomes, they will be reported to Dr. McLeod monthly.

Notification of an SAE or clinically significant AE must also be submitted to the Institutional Review Board (IRB) in accordance with its requirements. All Serious Ocular Adverse Events will be reported to the SCUT II/REAGIR DSMC on a monthly basis. All AEs must be reported from the time that the patient provides informed consent through the last study visit.

The study sponsor will be responsible for notifying the Food and Drug Administration (FDA) of any unexpected fatal or life-threatening suspected adverse reaction as soon as possible, but in no case later than 7 calendar days after the sponsor's initial receipt of the information.

### Reporting Events to Participants

AEs and SAEs will be discussed on an individual level with the masked treating physician. At the end of the study, all study participants will be given a report of the results in their native language.

### Events of Special Interest

Not applicable.

### Reporting of Pregnancy

Not applicable.

## Unanticipated Problems

### Definition of Unanticipated Problems (UP)

The Office for Human Research Protections (OHRP) considers unanticipated problems involving risks to participants or others to include, in general, any incident, experience, or outcome that meets **all** of the following criteria:

- Unexpected in terms of nature, severity, or frequency given (a) the research procedures that are described in the protocol-related documents, such as the Institutional Review Board (IRB)-approved research protocol and informed consent document; and (b) the characteristics of the participant population being studied;
- Related or possibly related to participation in the research (“possibly related” means there is a reasonable possibility that the incident, experience, or outcome may have been caused by the procedures involved in the research); and
- Suggests that the research places participants or others at a greater risk of harm (including physical, psychological, economic, or social harm) than was previously known or recognized.

### Unanticipated Problem Reporting

The investigator will report unanticipated problems (UPs) to the reviewing Institutional Review Board (IRB) and to the Data Coordinating Center (DCC)/lead principal investigator (PI). The UP report will include the following information:

- Protocol identifying information: protocol title and number, PI’s name, and the IRB project number;
- A detailed description of the event, incident, experience, or outcome;
- An explanation of the basis for determining that the event, incident, experience, or outcome represents an UP;
- A description of any changes to the protocol or other corrective actions that have been taken or are proposed in response to the UP.

To satisfy the requirement for prompt reporting, UPs will be reported using the following timeline:

- UPs that are serious adverse events (SAEs) will be reported to the IRB and to the DCC/study sponsor within 7 days of the investigator becoming aware of the event.
- Any other UP will be reported to the IRB and to the DCC/study sponsor within 30 days of the investigator becoming aware of the problem.
- All UPs should be reported to appropriate institutional officials (as required by an institution’s written reporting procedures), the supporting agency head (or designee), and the Office for Human Research Protections (OHRP) within 30 days of the IRB’s receipt of the report of the problem from the investigator.

### Reporting Unanticipated Problems to Participants

Participants will be notified of UPs in aggregate. When UPs affect an individual, they will be notified individually by the masked treating physician.

# STATISTICAL CONSIDERATIONS

## Statistical Hypotheses

**Specific Aim 1: To determine if corneal cross-linking with riboflavin (UVX) is a beneficial adjuvant in the treatment of smear- and/or culture-positive bacterial ulcers.**

1. We anticipate that UVX will result in better best spectacle corrected visual acuity (BSCVA) at 6 months compared with antibiotic alone.
2. We anticipate that UVX will result in faster microbiological cure rates, smaller scar size and lower rate of corneal perforation compared with antibiotic alone.

**Specific Aim 2: To determine if early topical steroids are a beneficial adjuvant in the treatment of smear- and/or culture-positive bacterial ulcers.**

1. We hypothesize that those randomized to early topical steroids will have improved BSCVA at 6 months compared with antibiotic alone.

**Specific Aim 3: To determine which ulcer characteristics predict the most benefit from the addition of adjuvant corneal cross-linking and/or early steroids.**

1. We hypothesize that culture positive bacterial ulcers with drug resistant organisms, as measured by MIC_50_, will benefit more from adjuvant UVX than those with antibiotic susceptible organisms.

**Specific Aim 4: To determine if cross linking with rose bengal (RB-PDT) is a beneficial adjuvant in the treatment of fungal, acanthamoeba, and smear or culture negative corneal ulcers.**

1. We hypothesize that there will be improved visual acuity at 6 months among those randomized to adjuvant RB-PDT after controlling for baseline visual acuity.
2. We anticipate that there will be smaller infiltrate/scar size and decreased rate of perforation and/or need for TPK at 6 months among RB-PDT treated patients.

Primary Endpoint: Best spectacle-corrected visual acuity at 6 months

Secondary Endpoints:

- Microbiological cure on repeat smear and culture at Day 2
- Interaction of minimum inhibitory concentration (MIC) and CXL on best spectacle-corrected visual acuity
- Infiltrate/scar size/depth, as measured by clinical exam, clinical photographs, Pentacam and OCT at week 3 and months 3, 6, and 12
- Adverse events including rate of perforation/need for TPK
- BSCVA at Day 1, 3 weeks, 3 months, and 12 months
- Astigmatism, higher order aberrations, topography, and densitometry as measured on Pentacam at week 3 and months 3, 6, and 12
- Corneal thickness and scar size/depth as measured by AS-OCT at week 3 and months 3, 6, and 12
- Basal nerve plexus, white blood cell count, and keratocyte density on confocal microscopy at week 3 and months 3, 6, and 12
- Visual function questionnaire (VFQ) will be compared between groups at 6 months, controlling for Day 1 VFQ
- Pain scale at baseline, Day 1, and Day 3
- Subgroup analysis of study participants receiving prior topical antimicrobial therapy

See **Sections 9.4.1 and 9.4.2** for prespecified statistical analyses for Primary and Secondary Endpoints.

## Sample Size Determination

**Sample Size Calculation for SCUT II Primary Analysis: BSCVA**. We power the study for each pairwise comparison. Specifically, we will be comparing (Specific Aim 1) CXL plus early steroids (arm 3) versus steroids and sham cross linking (arm 2), and (Specific Aim 2) early steroids with sham CXL (arm 2) versus standard therapy (arm 1). The power calculation was based on the primary outcome, 6-month BSCVA. We informed the calculation with measurements from the first Steroids for Corneal Ulcers Trial (SCUT), among patients enrolled with between 20/60 and 20/400 vision. The SCUT trial measured BSCVA at baseline, 3-months, and 12-months. We conservatively used the 12-month outcome measure for the calculations since there was no 6-month measurement. The standard deviation of BSCVA at 12 months was 0.293. Since the primary analysis will adjust for baseline BSCVA, we used an estimate of the residual standard deviation, which is ${SD}_{r}=SD\sqrt{1-r^{2}}$, where *r* is the correlation between the baseline measure and primary endpoint. In SCUT, the correlation between baseline and 12-month BSCVA among patients with between 20/60 and 20/400 vision at enrollment was 0.216. We thus assumed a residual standard deviation of $0.293\sqrt{1-{0.216}^{2}}=0.286$. Assuming a significance level of 0.05/2=0.025, and allowing for approximately 15% loss to follow up, we estimate that we will have 80% power to detect a 1.4-line difference (logMAR 0.14) between groups with 93 study participants per arm (279 total). This calculation applies to each of the two prespecified primary outcomes for this trial (corresponding to the two separate research questions), and is based on the standard power formula for the T-test (using an estimated residual standard deviation). Note that if the trial were to enroll 60 patients per arm, the minimum detectable effect under all the same assumptions would be 1.8 lines (logMAR 0.18). See SCUT II **SAP Section 1.5.6** for details on interim power analysis.

**SCUT II Sample Size Calculation for Secondary Outcome: Microbiological Cure**. Studies have suggested that in addition to providing an initial diagnosis, repeated culture can be used to assess response to treatment and is highly correlated with clinical outcomes such as visual acuity. We will re-culture all study participants 30 minutes after CXL or sham CXL (Day 2) to assess the effect of CXL on rate of microbiological cure. We hypothesize that those in the CXL group (Arm 3) will have a higher rate of microbiological cure on Day 2 cultures than those randomized to the Standard Therapy (Arm 1) or Early Steroid Group (Arm 2). Participants in both arms 1 and 2 will serve as the comparison group increasing the power in this analysis.

For microbiological outcomes, we will be comparing sham cross-linking to actual cross-linking, with specimens collected just prior to any administration of antibiotics (as discussed in the Manual of Operations). This comparison will be conducted contrasting Arm 3 to Arms 1 and 2 combined (which undergo sham cross-linking). We expect that approximately 70% of patients will show positive baseline cultures; only culture positive patients are likely to contribute information to the microbiological outcome. Taking into account loss to follow-up, we anticipate 80% power to detect at least a 26% difference in absolute terms between those experiencing cross linking (Arm 3) and those who did not receive cross linking (Arm 1 and Arm 2). The calculation assumes a comparison of two proportions. This is a prespecified secondary outcome.

Note that for both of these prespecified secondary analyses, the final hypothesis test will be conducted at an alpha of 0.025 (thereby applying a Bonferroni correction for two hypothesis tests).

**SCUT II Effects Stratified by Presence of Drug resistant Organisms**. This analysis is similar to the microbiological outcome in the secondary analyses, in that we contrast culture-positive patients from Arm 3 to culture-positive patients from Groups 1 and 2 (Specific Aim 3). Prior studies suggest a drug resistance to moxifloxacin probability of between 0.34 and 0.43, depending on prior exposure to moxifloxacin. We hypothesize that when drug resistant organisms are present, outcomes will be more favorable when cross-linking is conducted. The outcome variable is visual acuity. We anticipate approximately 80% power to detect a difference of approximately 3 lines in outcomes, assuming 35% of individuals are drug resistant at baseline (based on simulation; details available upon request). The calculation assumed 15% loss to follow-up. This is a prespecified secondary analysis.

See **Section 9.4.1** for details regarding pre-specified analyses.

**REAGIR Primary outcome, BSCVA at 6 months**. The sample size calculation for REAGIR used the same assumptions as the SCUT II sample size calculation for BSCVA. Assuming a significance level of 0.05, allowing for approximately 15% loss to follow up, we estimate that we will have 90% power to detect a 1.1-line difference (logMAR 0.11) between groups with 165 study participants per arm (330 total). For the same sample size and under the same assumptions, the detectable difference at 80% power is 1.0-lines (logMAR 0.10). These calculations were based on the standard power formula for the T-test (using an estimated residual standard deviation).

## Populations for Analyses

Our analysis will adhere strictly to the intent-to-treat principle. All participants randomized will be analyzed according to their allocation.

**Missing Outcomes:** We distinguish two kinds of missing data: missing data in covariates, and missing data for outcomes. For the latter, we distinguish simple loss to follow-up (as when a patient has needed to move out of the area) from data missing because of the occurrence of a medical event (such as the loss of an eye). We will always report the results of complete case analysis, although such results are known to be biased.[*^82^*](#_ENREF_82) In the event of missing baseline covariates among >10% of patients, we will use regression based multiple imputation (with at least 100 replicates) to impute these missing covariates (based only on other variables available at baseline). To compare with the complete case analysis, we will also report multiple-imputation based analysis for missing outcomes (or equivalently, likelihood methods which integrate over the missing outcome data based on the same assumption of missingness at random); such analyses are important to report because of the intent to treat principle. Finally, we conduct sensitivity analyses in which the data are not assumed missing at random, to assess how extreme the missing values would need to be to change the conclusions of the study. We propose use of the R package mice (Multiple Imputation by Chained Equations) for multiple imputation; other choices are available and would serve equally well.

## Statistical Analyses

### Analysis of the Primary Efficacy Endpoint

The **Statistical Analysis Plan** for SCUT II and REAGIR include more detail about planned analyses. The primary outcome will be 6-month BSCVA, and the primary analysis of each trial will use the same approach. We will use multiple linear regression models to evaluate BSCVA measured at 6 months with covariates for treatment arm (expressed as a binary indicator variable for steroids and a binary indicator variable for cross-linking), indicators for study site (randomization strata), and BSCVA measured on Day 1 (before treatment). We propose a two-sided alpha of 0.025 for each pairwise primary comparison. Specifically, we propose to contrast Groups 1 and 2 using a linear model in which we use baseline visual acuity and a binary indicator for assignment to Group 2 as predictors, and BSCVA as the outcome variable. We then propose to contrast Groups 2 and 3 separately using a model with baseline visual acuity and a binary indicator for assignment to Group 3 as predictors, with BSCVA as the outcome variable.

For study participants who experience perforation or the need for TPK this will be noted and BSCVA will be recorded prior to performing surgery (either corneal glue or TPK). The last observation (at 3 weeks or 3 months) will be carried forward (LOCF) as the 6-month BSCVA, but a separate secondary analysis will include these study participants actual 6-month visual acuity.

A supplementary analysis using linear mixed effects regression will be conducted to analyze all BSCVA data (including 3-week and 3-month observations). The model will include baseline BSCVA and treatment assignment as covariates (fixed effects) plus a random effect for patient to allow for repeated measures over time. Such analyses will be sharply distinguished from the primary pre-specified analysis. Permutation testing will be the basis of inference. Specifically, we will conduct 10,000 random permutations of treatment assignment, and conduct the primary regression analysis on these re-randomized data sets. We will then compare the observed regression coefficients to the randomization distribution, reporting the quantiles.

### Analysis of the Secondary Endpoints

**Microbiological cure.** Studies have suggested that in addition to providing an initial diagnosis, repeated culture can be used to assess response to treatment and is highly correlated with clinical outcomes such as visual acuity.[^83-86^](#_ENREF_83) We will re-culture all study participants at Day 2, prior to starting antibiotics to assess the effect of CXL on rate of microbiological cure. We hypothesize that those in the CXL group (Group 3) will have a higher rate of microbiological cure on Day 2 cultures than those randomized to the Standard Therapy (Group 1) or Early Steroid Group (Group 2). Participants in both group 1 & 2 will serve as the comparison group increasing the power in this analysis.

We propose the primary analysis to be a Fisher’s exact test comparing the proportion of positivity at follow-up between initially culture positive individuals who were assigned to cross linking (Group 3) versus initially culture positive individuals assigned to sham cross linking (Groups 1 and 2). Additionally, we will report the results for initially culture negative individuals as a supplementary analysis in a logistic regression with assignment, indicators for site (randomization strata), and initial culture results as covariates.

**Scar/infiltrate.** The analysis for scar/infiltrate size will follow the templates for visual acuity given above. Multiple linear regression models will be used to evaluate 12-month scar size by treatment arm (a 3-level categorical variable) while correcting for baseline measurements. As in the primary analysis, In SCUT II (groups 1, 2, 3) we will perform pairwise comparisons between arms with a significance level of 0.05/2=0.025. In REAGIR (groups 4, 5) we will compare the two arms with significance level of 0.05. Corneal thinning and scarring will be evaluated similarly using Anterior Segment Optical Coherence Tomography (AS-OCT) correcting for baseline values.

**Corneal Perforation.** A Cox proportional hazards model will estimate the hazard of perforation, defined as perforation (flat anterior chamber with presence of iris plugging a defect in the cornea or seidel positivity) or the need for TPK while correcting for baseline infiltrate depth.

**Visual Function Questionnaire (VFQ).** VFQ will be compared between groups controlling for Day 1 VFQ. NEI-VFQ in the US and the Indian-VFQ (IND-VFQ) in India. This will be conducted using linear regression with baseline and assignment variables.

### Safety Analyses

Interim reports for the DSMC will be prepared by the Data Coordinating Center at Proctor. These reports will include (a) recruitment overall, and by study site, (b) compliance, and (c) retention. The reports will also list study outcomes, including 6-month BSCVA and microbiological outcomes, and all adverse outcomes, including mortality and perforations. See **Sections 9.4.1** and **9.4.2** of this protocol for a discussion of primary and secondary analyses to be performed.

All adverse events will be tabulated and reported. Statistical comparisons will be conducted using Fisher’s exact test, but with the caution that failure to find a statistically significant difference cannot be used to infer a lack of risk difference, since the study is not powered to examine rare outcomes. Procedures for reporting both adverse events and serious adverse events, including notification of the Medical Monitor, will be reviewed by the DSMC prior to opening enrollment. We will categorize adverse events, severe adverse events and events of interest following recommended best practices for clinical trial monitoring and reporting. The **Statistical Analysis Plan – Section 1.5.6 (SCUT II) and Section 3.5 (REAGIR)** include details about interim analyses and stopping guidelines.[^87^](#_ENREF_87)

# SUPPORTING DOCUMENTATION AND OPERATIONAL CONSIDERATIONS

## Regulatory, Ethical, and Study Oversight Considerations

### Informed Consent Process

#### Consent/assent and Other Informational Documents Provided to participants

Consent forms describing in detail the study intervention, study procedures, and risks are given to the participant and written documentation of informed consent is required prior to starting intervention/administering study intervention. Consent forms will be given to the participant in English or Spanish (US sites), Tamil (India sites), or Portuguese (Brazil site).

#### Consent Procedures and Documentation

Informed consent is a process that is initiated prior to the individual’s agreeing to participate in the study and continues throughout the individual’s study participation. Consent forms will be Institutional Review Board (IRB)-approved and the participant will be asked to read and review the document. The investigator will explain the research study to the participant and answer any questions that may arise. A verbal explanation will be provided in terms suited to the participant’s comprehension of the purposes, procedures, and potential risks of the study and of their rights as research participants. Participants will have the opportunity to carefully review the written consent form and ask questions prior to signing. The participants should have the opportunity to discuss the study with their family or surrogates or think about it prior to agreeing to participate. The participant will sign the informed consent document prior to any procedures being done specifically for the study. Participants must be informed that participation is voluntary and that they may withdraw from the study at any time, without prejudice. A copy of the informed consent document will be given to the participants for their records. The informed consent process will be conducted and documented in the source document (including the date), and the form signed, before the participant undergoes any study-specific procedures. The rights and welfare of the participants will be protected by emphasizing to them that the quality of their medical care will not be adversely affected if they decline to participate in this study.

### Study Discontinuation and Closure

This study may be temporarily suspended or prematurely terminated if there is sufficient reasonable cause. Written notification, documenting the reason for study suspension or termination, will be provided by the suspending or terminating party to study participants, investigator, funding agency, sponsor, and regulatory authorities. The **Statistical Analysis Plan – Section 1.5.6** includes details of interim stopping rules based on efficacy or futility. If the study is prematurely terminated or suspended, the Principal Investigator (PI) will promptly inform study participants, the Institutional Review Board (IRB), and sponsor and will provide the reason(s) for the termination or suspension. Study participants will be contacted, as applicable, and be informed of changes to study visit schedule.

Circumstances that may warrant termination or suspension include, but are not limited to:

- Determination of unexpected, significant, or unacceptable risk to participants
- Demonstration of efficacy that would warrant stopping
- Insufficient compliance to protocol requirements
- Data that are not sufficiently complete and/or evaluable
- Determination that the primary endpoint has been met
- Determination of futility

Study may resume once concerns about safety, protocol compliance, and data quality are addressed, and satisfy the sponsor, IRB and/or Food and Drug Administration (FDA).

### Confidentiality and Privacy

Participant confidentiality and privacy is strictly held in trust by the participating investigators, their staff, and the sponsor(s) and their interventions. This confidentiality is extended to cover testing of biological samples and genetic tests in addition to the clinical information relating to participants. Therefore, the study protocol, documentation, data, and all other information generated will be held in strict confidence. No information concerning the study or the data will be released to any unauthorized third party without prior written approval of the sponsor.

All research activities will be conducted in as private a setting as possible.

The study monitor, other authorized representatives of the sponsor, representatives of the Institutional Review Board (IRB), regulatory agencies or pharmaceutical company supplying study product may inspect all documents and records required to be maintained by the investigator, including but not limited to, medical records (office, clinic, or hospital) and pharmacy records for the participants in this study. The clinical study site will permit access to such records.

The study participant’s contact information will be securely stored at each clinical site for internal use during the study. At the end of the study, all records will continue to be kept in a secure location for as long a period as dictated by the reviewing IRB, Institutional policies, or sponsor requirements.

Study participant research data, which is for purposes of statistical analysis and scientific reporting, will be transmitted to and stored at the Data Coordinating Center. This will not include the participant’s contact or identifying information. Rather, individual participants and their research data will be identified by a unique study identification number. The study data entry and study management systems used by clinical sites and by the Data Coordinating Center research staff will be secured and password protected. At the end of the study, all study databases will be de-identified and archived at the Data Coordinating Center at UCSF.

Certificate of Confidentiality

To further protect the privacy of study participants, a Certificate of Confidentiality will be issued by the National Institutes of Health (NIH). This certificate protects identifiable research information from forced disclosure. It allows the investigator and others who have access to research records to refuse to disclose identifying information on research participation in any civil, criminal, administrative, legislative, or other proceeding, whether at the federal, state, or local level. By protecting researchers and institutions from being compelled to disclose information that would identify research participants, Certificates of Confidentiality help achieve the research objectives and promote participation in studies by helping assure confidentiality and privacy to participants.

### Future Use of Stored Specimens and Data

Data collected for this study will be analyzed and stored at the Data Coordinating Center at UCSF. After the study is completed, the de-identified, archived data will be transmitted to and stored on the Research Electronic Data Capture system (REDCap).

With the participant’s approval and as approved by local Institutional Review Boards (IRBs), de-identified biological samples will be stored at the enrollment site. These samples could be used to research the relationship between minimum inhibitory concentration and clinical outcomes, as well as drug resistance.

During the conduct of the study, an individual participant can choose to withdraw consent to have biological specimens stored for future research. However, withdrawal of consent with regard to biosample storage may not be possible after the study is completed.

When the study is completed, access to study data will be provided through REDCap.

### Key Roles and Study Governance

| **Co-Principal Investigator** | **Co-Principal Investigator** | **Medical Monitor** |
| --- | --- | --- |
| Tom Lietman, MD  Director and Professor | Jennifer Rose-Nussbaumer, MD | Stephen McLeod, MD  Chair and Professor |
| Francis I. Proctor Foundation, University of California, San Francisco | Stanford University | Department of Ophthalmology, University of California, San Francisco |
| 490 Illinois Street  San Francisco, CA 94158 | 2452 Watson Court  Palo Alto CA 94303 | 490 Illinois Street  San Francisco, CA 94158 |
| 415-502-2662 | 650-722-7422 | 415-476-1922 |
| Tom.Lietman@ucsf.edu | rosej@stanford.edu | Stephen.McLeod@ucsf.edu |

Please see **MOP Section 2** for detailed descriptions of study investigators and staff.

### Safety Oversight

A Data and Safety Monitoring Committee (DSMC) has been empaneled by the NEI. The committee consists of six individuals, including cornea specialists; an independent biostatistician; and a bioethicist. The committee will meet in person at least once per year and will convene biannual teleconferences for progress reports. *Ad hoc* meetings as needed may also be convened. All study protocols will be subject to review and approval by Institutional Review Boards. Please refer to the **Statistical Analysis Plan – Section 1.5.6** for a detailed description of our planned interim analyses.

### Clinical Monitoring

Clinical site monitoring is conducted to ensure that the rights and well-being of trial participants are protected, that the reported trial data are accurate, complete, and verifiable, and that the conduct of the trial is in compliance with the currently approved protocol/amendment(s), with International Conference on Harmonisation Good Clinical Practice (ICH GCP), and with applicable regulatory requirement(s).

Clinical monitoring will be conducted by the Clinical Coordinating Center (CCC) at Stanford University. A two-person team will make site visits to all enrollment sites three times per year to monitor study activities. At each visit, the CCC will check the quality of photographs, sit in refractions to ensure the proper refraction protocol is followed, and visit the microbiology lab. During each visit the CCC will conduct a complete chart review of all patient charts to ensure data is being recorded in a complete fashion. The Data Coordinating Center will conduct regular weekly off-site reviews of data entered in REDCap to ensure 100% data verification.

### Quality Assurance and Quality Control

Each clinical site will perform internal quality management of study conduct, data and biological specimen collection, documentation and completion. An individualized quality management plan will be developed to describe a site’s quality management.

Quality control (QC) procedures will be implemented beginning with the data entry system and data QC checks that will be run on the database will be generated. Any missing data or data anomalies will be communicated to the site(s) for clarification/resolution.

Following written Standard Operating Procedures (SOPs), the monitors will verify that the clinical trial is conducted and data are generated and biological specimens are collected, documented (recorded), and reported in compliance with the protocol, International Conference on Harmonisation Good Clinical Practice (ICH GCP), and applicable regulatory requirements (e.g., Good Laboratory Practices (GLP), Good Manufacturing Practices (GMP)).

The investigational site will provide direct access to all trial related sites, source data/documents, and reports for the purpose of monitoring and auditing by the sponsor, and inspection by local and regulatory authorities.

### Data Handling and Record Keeping

#### Data Collection and Management Responsibilities

Data collection is the responsibility of the clinical trial staff at the site under the supervision of the site investigator. The investigator is responsible for ensuring the accuracy, completeness, legibility, and timeliness of the data reported.

Clinical data (including adverse events (AEs), concomitant medications, and expected adverse reactions data) and clinical laboratory data will be entered into Research Electronic Data Capture (REDCap), a 21 CFR Part 11-compliant data capture system provided by the Data Coordinating Center at UCSF. The data system includes password protection and internal quality checks, such as automatic range checks, to identify data that appear inconsistent, incomplete, or inaccurate.

Any hardcopies of the study visit worksheets will be provided for use as source document worksheets

for recording data for each participant enrolled in the study. All source documents should be completed in a neat, legible manner to ensure accurate interpretation of data. Clinical data will be double data-entered directly from the source documents.

#### Study Records Retention

Study documents should be retained for a minimum of 2 years after the last approval of a marketing application in an International Conference on Harminosation (ICH) region and until there are no pending or contemplated marketing applications in an ICH region or until at least 2 years have elapsed since the formal discontinuation of clinical development of the study intervention. These documents should be retained for a longer period, however, if required by local regulations. No records will be destroyed without the written consent of the sponsor, if applicable. It is the responsibility of the sponsor to inform the investigator when these documents no longer need to be retained.

### Protocol Deviations

A protocol deviation is any noncompliance with the clinical trial protocol, ICH GCP, or MOP requirements. The noncompliance may be either on the part of the participant, the investigator, or the study site staff. As a result of deviations, corrective actions are to be developed by the site and implemented promptly.

These practices are consistent with ICH GCP:

- 4.5 Compliance with Protocol, sections 4.5.1, 4.5.2, and 4.5.3
- 5.1 Quality Assurance and Quality Control, section 5.1.1
- 5.20 Noncompliance, sections 5.20.1, and 5.20.2.

It is the responsibility of the site investigator to use continuous vigilance to identify and report deviations within 7 working days of identification of the protocol deviation, or within 7 working days of the scheduled protocol-required activity. All deviations must be addressed in study source documents, reported to National Eye Institute Program Official and the Coordinating Center at Stanford. Protocol deviations must be sent to the reviewing Institutional Review Board (IRB) per their policies. The site investigator is responsible for knowing and adhering to the reviewing IRB requirements.

### Publication and Data Sharing Policy

This study will comply with the NIH Data Sharing Policy and Policy on the Dissemination of NIH-Funded Clinical Trial Information and the Clinical Trials Registration and Results Information Submission rule. As such, this trial will be registered at ClinicalTrials.gov, and results from this trial will be submitted and published on ClinicalTrials.gov. In addition, every attempt will be made to publish results in peer-reviewed journals. Consistent with the collaborative nature of the proposed research, the PI anticipates sharing all data generated by the study with collaborators. Analytic datasets that will be developed through the project will comply with the NIH Data Sharing Policy. The analytical datasets from this project will include patient-level data generated from the study visits.

This study will adhere to the National Institutes of Health (NIH) Public Access Policy, which ensures that the public has access to the published results of NIH funded research. It requires scientists to submit final peer-reviewed journal manuscripts that arise from NIH funds to the digital archive PubMed Central upon acceptance for publication. External investigators can contact the study PIs to initiate a request for study data to support new study proposals or manuscripts. Approval of such requests and initiation of collaborations will consider the following criteria:

1. The proposed project must be of high scientific merit.
2. The proposed project must be consistent with the overall goals and objectives of the parent study.
3. The proposed ancillary project must meet certain participant burden criteria (for any new primary data collection involving subjects), including:
   1. Acceptable to the subjects (e.g., risks, time, discomfort, privacy); and,
   2. Not hinder or disrupt clinical care provided by study sites.
4. The proposed project’s investigators must plan for adequate resources to effectively complete the project, including:
   1. Sufficient budget to cover costs of personnel and supplies; and
   2. Staff possessing the requisite expertise to meet the objectives of the project.
5. The proposed project should document any involvement of parent study investigators as part of the research team.

Approved requests for data will follow data sharing agreements that UCSF has with NIH. Data will be de-identified prior to release for sharing. However, there remains the possibility of deductive disclosure of subjects with unusual characteristics and disclosure of UCSF proprietary information. Thus, researchers who seek access to individual level data will be required to sign a data sharing agreement prior to release for sharing. The agreement provides for: (1) a commitment to using the data for research purposes only and not to identify any individual participants or to disclose proprietary information; (2) a commitment to securing the data using appropriate computer technology; (3) a commitment to destroying or returning the data after analyses are completed; and (4) a commitment to meet any requirements that might be stipulated by the IRB at UCSF.

### Conflict of Interest Policy

The independence of this study from any actual or perceived influence, such as by the pharmaceutical industry, is critical. Therefore, any actual conflict of interest of persons who have a role in the design, conduct, analysis, publication, or any aspect of this trial will be disclosed and managed. Furthermore, persons who have a perceived conflict of interest will be required to have such conflicts managed in a way that is appropriate to their participation in the design and conduct of this trial. The study leadership in conjunction with the National Eye Institute has established policies and procedures for all study group members to disclose all conflicts of interest and will establish a mechanism for the management of all reported dualities of interest.

## Additional Considerations

None

## Abbreviations

| AE | Adverse Event |
| --- | --- |
| AS-OCT | Anterior Segment Optical Coherence Tomography |
| BSCVA | Best Spectacle-Corrected Visual Acuity |
| CFR | Code of Federal Regulations |
| CCC | Clinical Coordinating Center |
| CONSORT | Consolidated Standards of Reporting Trials |
| CXL | Corneal Cross-Linking |
| DCC | Data Coordinating Center |
| DSMC | Data Safety Monitoring Committee |
| ETDRS | Early Treatment Diabetic Retinopathy Study |
| FDA | Food and Drug Administration |
| GCP | Good Clinical Practice |
| GLP | Good Laboratory Practices |
| GMP | Good Manufacturing Practices |
| ICH | International Conference on Harmonisation |
| IDE | Investigational Device Exemption |
| IOP | Intraocular Pressure |
| IND | Investigational New Drug Application |
| IND-VFQ | Indian Visual Function Questionnaire |
| IOP | Intraocular Pressure |
| IRB | Institutional Review Board |
| LASIK | Laser-Assisted in situ Keratomileusis |
| LOCF | Last Observation Carried Forward |
| logMAR | Logarithm of the Minimum Angle |
| MIC | Minimum Inhibitory Concentration |
| MOP | Manual of Operations and Procedures |
| MRSA | Methicillin-Resistant Staphylococcus Aureus |
| MRx | Manifest Refraction |
| NCT | National Clinical Trial |
| NEI | National Eye Institute |
| NEI-VFQ | National Eye Institute Visual Function Questionnaire |
| NIH | National Institutes of Health |
| NSAE | Non-Serious Adverse Event |
| OCT | Optical Coherence Tomography |
| OHRP | Office for Human Research Protections |
| PI | Principal Investigator |
| PKP | Penetrating Keratoplasty |
| PRC | Proctor Reading Center |
| QA | Quality Assurance |
| QC | Quality Control |
| QID | Four times daily |
| RB | Rose Bengal |
| RB-PDT | Rose Bengal Photodynamic Therapy |
| REAGIR | Rose Bengal Electromagnetic Activation with Green light for Infection Reduction |
| REDCap | Research Electronic Data Capture |
| SAE | Serious Adverse Event |
| SAP | Statistical Analysis Plan |
| SCUT | Steroids for Corneal Ulcer Trial |
| SCUT II | Steroids and Cross-linking for Ulcer Treatment |
| SoA | Schedule of Activities |
| SOP | Standard Operating Procedure |
| TID | Three times daily |
| TPK | Therapeutic Penetrating Keratoplasty |
| UCSF | University of California, San Francisco |
| UNIFESP | Federal University of São Paulo |
| UP | Unanticipated Problem |
| US | United States |
| UV | Ultra-violet |
| UV-A | Ultra-violet A |
| UVX | Corneal Cross-Linking with riboflavin |
| VFQ | Visual Function Questionnaire |

## Protocol Amendment History

The table below is intended to capture changes of IRB-approved versions of the protocol, including a description of the change and rationale. A Summary of Changes table for the current amendment is located in the Protocol Title Page.

| **Version** | **Date** | **Description of Change** | **Brief Rationale** |
| --- | --- | --- | --- |
| 2.0 | 17 July 2020 | Addition of Specific Aim 4 |  |
| 2.0 | 21 July 2021 | Added detail about baseline information collected/entered in REDCap for Rancho Park Compounding Pharmacy | A pharmacist at Rancho Park will review current medications and any drug allergies prior to dispensing study medication. Patient addresses are needed for shipment of study medication directly to participants. |
| 2.0 | 23 February 2022 | Clarification that patients randomized to Groups 1 & 2 receive saline drops during UVX |  |
|  |  |  |  |

# REFERENCES

1. McDonald EM, Ram FS, Patel DV, McGhee CN. Topical antibiotics for the management of bacterial keratitis: an evidence-based review of high quality randomised controlled trials. *The British journal of ophthalmology.* 2014;98(11):1470-1477.

2. Tan DT, Janardhanan P, Zhou H, et al. Penetrating keratoplasty in Asian eyes: the Singapore Corneal Transplant Study. *Ophthalmology.* 2008;115(6):975-982 e971.

3. Hobden JA. Pseudomonas aeruginosa proteases and corneal virulence. *DNA and cell biology.* 2002;21(5-6):391-396.

4. Matsumoto K. Role of bacterial proteases in pseudomonal and serratial keratitis. *Biological chemistry.* 2004;385(11):1007-1016.

5. Ray KJ, Srinivasan M, Mascarenhas J, et al. Early addition of topical corticosteroids in the treatment of bacterial keratitis. *JAMA ophthalmology.* 2014;132(6):737-741.

6. Palioura S, Henry CR, Amescua G, Alfonso EC. Role of steroids in the treatment of bacterial keratitis. *Clinical ophthalmology.* 2016;10:179-186.

7. Alio JL, Abbouda A, Valle DD, Del Castillo JM, Fernandez JA. Corneal cross linking and infectious keratitis: a systematic review with a meta-analysis of reported cases. *Journal of ophthalmic inflammation and infection.* 2013;3(1):47.

8. Sand D, She R, Shulman IA, Chen DS, Schur M, Hsu HY. Microbial keratitis in los angeles: the doheny eye institute and the los angeles county hospital experience. *Ophthalmology.* 2015;122(5):918-924.

9. Papaioannou L, Miligkos M, Papathanassiou M. Corneal Collagen Cross-Linking for Infectious Keratitis: A Systematic Review and Meta-Analysis. *Cornea.* 2016;35(1):62-71.

10. Martins SA, Combs JC, Noguera G, et al. Antimicrobial efficacy of riboflavin/UVA combination (365 nm) in vitro for bacterial and fungal isolates: a potential new treatment for infectious keratitis. *Investigative ophthalmology & visual science.* 2008;49(8):3402-3408.

11. Panda A, Krishna SN, Kumar S. Photo-activated riboflavin therapy of refractory corneal ulcers. *Cornea.* 2012;31(10):1210-1213.

12. Iseli HP, Thiel MA, Hafezi F, Kampmeier J, Seiler T. Ultraviolet A/riboflavin corneal cross-linking for infectious keratitis associated with corneal melts. *Cornea.* 2008;27(5):590-594.

13. Makdoumi K, Mortensen J, Crafoord S. Infectious keratitis treated with corneal crosslinking. *Cornea.* 2010;29(12):1353-1358.

14. Shetty R, Nagaraja H, Jayadev C, Shivanna Y, Kugar T. Collagen crosslinking in the management of advanced non-resolving microbial keratitis. *The British journal of ophthalmology.* 2014;98(8):1033-1035.

15. Makdoumi K, Mortensen J, Sorkhabi O, Malmvall BE, Crafoord S. UVA-riboflavin photochemical therapy of bacterial keratitis: a pilot study. *Graefe's archive for clinical and experimental ophthalmology = Albrecht von Graefes Archiv fur klinische und experimentelle Ophthalmologie.* 2012;250(1):95-102.

16. Zhu H, Alt C, Webb RH, Melki S, Kochevar IE. Corneal Crosslinking With Rose Bengal and Green Light: Efficacy and Safety Evaluation. *Cornea.* 2016;35(9):1234-1241.

17. Lorenzo-Martin E, Gallego-Munoz P, Ibares-Frias L, et al. Rose Bengal and Green Light Versus Riboflavin-UVA Cross-Linking: Corneal Wound Repair Response. *Invest Ophthalmol Vis Sci.* 2018;59(12):4821-4830.

18. Cherfan D, Verter EE, Melki S, et al. Collagen cross-linking using rose bengal and green light to increase corneal stiffness. *Invest Ophthalmol Vis Sci.* 2013;54(5):3426-3433.

19. Bekesi N, Gallego-Munoz P, Ibares-Frias L, et al. Biomechanical Changes After In Vivo Collagen Cross-Linking With Rose Bengal-Green Light and Riboflavin-UVA. *Invest Ophthalmol Vis Sci.* 2017;58(3):1612-1620.

20. Wang T, Zhu L, Zhu J, et al. Subacute effects of rose Bengal/Green light cross linking on rabbit thin corneal stability and safety. *Lasers Surg Med.* 2018;50(4):324-332.

21. Naranjo A, Pelaez D, Arrieta E, et al. Cellular and molecular assessment of rose bengal photodynamic antimicrobial therapy on keratocytes, corneal endothelium and limbal stem cell niche. *Exp Eye Res.* 2019;188:107808.

22. Gallego-Munoz P, Ibares-Frias L, Lorenzo E, et al. Corneal Wound Repair After Rose Bengal and Green Light Crosslinking: Clinical and Histologic Study. *Invest Ophthalmol Vis Sci.* 2017;58(9):3471-3480.

23. Prajna NV, Radhakrishnan N, Lalitha P, et al. Cross-Linking-Assisted Infection Reduction: A Randomized Clinical Trial Evaluating the Effect of Adjuvant Cross-Linking on Outcomes in Fungal Keratitis. *Ophthalmology.* 2019.

24. Atalay HT, Dogruman-Al F, Sarzhanov F, et al. Effect of Riboflavin/Rose Bengal-Mediated PACK-CXL on Acanthamoeba Trophozoites and Cysts in Vitro. *Curr Eye Res.* 2018;43(11):1322-1325.

25. Arboleda A, Miller D, Cabot F, et al. Assessment of rose bengal versus riboflavin photodynamic therapy for inhibition of fungal keratitis isolates. *Am J Ophthalmol.* 2014;158(1):64-70.e62.

26. Ozbek-Uzman S, Yalniz-Akkaya Z, Burcu A. Corneal Collagen Cross-Linking With Photoactivated Chromophore for Infectious Keratitis After Penetrating Keratoplasty. *Cornea.* 2020;39(3):283-289.

27. Kasetsuwan N, Reinprayoon U, Satitpitakul V. Photoactivated Chromophore for Moderate to Severe Infectious Keratitis as an Adjunct Therapy: A Randomized Controlled Trial. *Am J Ophthalmol.* 2016;165:94-99.

28. Said DG, Elalfy MS, Gatzioufas Z, et al. Collagen cross-linking with photoactivated riboflavin (PACK-CXL) for the treatment of advanced infectious keratitis with corneal melting. *Ophthalmology.* 2014;121(7):1377-1382.

29. Arafat SN, Robert MC, Shukla AN, Dohlman CH, Chodosh J, Ciolino JB. UV cross-linking of donor corneas confers resistance to keratolysis. *Cornea.* 2014;33(9):955-959.

30. Fadlallah A, Zhu H, Arafat S, Kochevar I, Melki S, Ciolino JB. Corneal Resistance to Keratolysis After Collagen Crosslinking With Rose Bengal and Green Light. *Invest Ophthalmol Vis Sci.* 2016;57(15):6610-6614.

31. Ortega-Usobiaga J, Llovet-Osuna F, Djodeyre MR, Llovet-Rausell A, Beltran J, Baviera J. Incidence of corneal infections after laser in situ keratomileusis and surface ablation when moxifloxacin and tobramycin are used as postoperative treatment. *Journal of cataract and refractive surgery.* 2015;41(6):1210-1216.

32. Ni N, Nam EM, Hammersmith KM, et al. Seasonal, geographic, and antimicrobial resistance patterns in microbial keratitis: 4-year experience in eastern Pennsylvania. *Cornea.* 2015;34(3):296-302.

33. Gupta N, Tandon R, Gupta SK, Sreenivas V, Vashist P. Burden of corneal blindness in India. *Indian journal of community medicine : official publication of Indian Association of Preventive & Social Medicine.* 2013;38(4):198-206.

34. Gonzales CA, Srinivasan M, Whitcher JP, Smolin G. Incidence of corneal ulceration in Madurai district, South India. *Ophthalmic epidemiology.* 1996;3(3):159-166.

35. Whitcher JP, Srinivasan M. Corneal ulceration in the developing world--a silent epidemic. *The British journal of ophthalmology.* 1997;81(8):622-623.

36. Pascolini D, Mariotti SP. Global estimates of visual impairment: 2010. *The British journal of ophthalmology.* 2012;96(5):614-618.

37. Whitcher JP, Srinivasan M, Upadhyay MP. Corneal blindness: a global perspective. *Bulletin of the World Health Organization.* 2001;79(3):214-221.

38. 2013 ABKP. Bacterial Keratitis Preferred Practice Pattern (PPP) Guideline. In: American Academy of Ophthalmology; 2013.

39. Jeng BH, Gritz DC, Kumar AB, et al. Epidemiology of ulcerative keratitis in Northern California. *Archives of ophthalmology.* 2010;128(8):1022-1028.

40. Thylefors B, Negrel AD, Pararajasegaram R, Dadzie KY. Global data on blindness. *Bulletin of the World Health Organization.* 1995;73(1):115-121.

41. Rose-Nussbaumer J, Prajna NV, Krishnan KT, et al. Vision-Related Quality-of-Life Outcomes in the Mycotic Ulcer Treatment Trial I: A Randomized Clinical Trial. *JAMA ophthalmology.* 2015;133(6):642-646.

42. Austin A, Schallhorn J, Geske M, Mannis M, Lietman T, Rose-Nussbaumer J. Empirical treatment of bacterial keratitis: an international survey of corneal specialists. *BMJ Open Ophthalmol.* 2017;2.

43. Prajna NV, Radhakrishnan N, Lalitha P, et al. Cross-Linking-Assisted Infection Reduction: A Randomized Clinical Trial Evaluating the Effect of Adjuvant Cross-Linking on Outcomes in Fungal Keratitis. *Ophthalmology.* 2020;127(2):159-166.

44. Narayana S, Krishnan T, Ramakrishnan S, et al. Mycotic Antimicrobial Localized Injection: A Randomized Clinical Trial Evaluating Intrastromal Injection of Voriconazole. *Ophthalmology.* 2019;126(8):1084-1089.

45. O'Day DM, Head WS, Robinson RD, Clanton JA. Corneal penetration of topical amphotericin B and natamycin. *Current eye research.* 1986;5(11):877-882.

46. Dart JK, Saw VP, Kilvington S. Acanthamoeba keratitis: diagnosis and treatment update 2009. *Am J Ophthalmol.* 2009;148(4):487-499.e482.

47. Papa V, Rama P, Radford C, Minassian DC, Dart JKG. Acanthamoeba keratitis therapy: time to cure and visual outcome analysis for different antiamoebic therapies in 227 cases. *Br J Ophthalmol.* 2019.

48. Carrijo-Carvalho LC, Sant'ana VP, Foronda AS, de Freitas D, de Souza Carvalho FR. Therapeutic agents and biocides for ocular infections by free-living amoebae of Acanthamoeba genus. *Surv Ophthalmol.* 2017;62(2):203-218.

49. Acharya NR, Srinivasan M, Mascarenhas J, et al. The steroid controversy in bacterial keratitis. *Archives of ophthalmology.* 2009;127(9):1231.

50. Cohen EJ. The case against the use of steroids in the treatment of bacterial keratitis. *Archives of ophthalmology.* 2009;127(1):103-104.

51. Hindman HB, Patel SB, Jun AS. Rationale for adjunctive topical corticosteroids in bacterial keratitis. *Archives of ophthalmology.* 2009;127(1):97-102.

52. Den S, Sotozono C, Kinoshita S, Ikeda T. Efficacy of early systemic betamethasone or cyclosporin A after corneal alkali injury via inflammatory cytokine reduction. *Acta ophthalmologica Scandinavica.* 2004;82(2):195-199.

53. Yi K, Chung TY, Hyon JY, Koh JW, Wee WR, Shin YJ. Combined treatment with antioxidants and immunosuppressants on cytokine release by human peripheral blood mononuclear cells - chemically injured keratocyte reaction. *Molecular vision.* 2011;17:2665-2671.

54. Williams RN, Paterson CA. The influence of topical corticosteroid therapy upon polymorphonuclear leukocyte distribution, vascular integrity and ascorbate levels in endotoxin-induced inflammation of the rabbit eye. *Experimental eye research.* 1987;44(2):191-198.

55. Chung JH, Kang YG, Kim HJ. Effect of 0.1% dexamethasone on epithelial healing in experimental corneal alkali wounds: morphological changes during the repair process. *Graefe's archive for clinical and experimental ophthalmology = Albrecht von Graefes Archiv fur klinische und experimentelle Ophthalmologie.* 1998;236(7):537-545.

56. Tomas-Barberan S, Fagerholm P. Influence of topical treatment on epithelial wound healing and pain in the early postoperative period following photorefractive keratectomy. *Acta ophthalmologica Scandinavica.* 1999;77(2):135-138.

57. Gritz DC, Kwitko S, Trousdale MD, Gonzalez VH, McDonnell PJ. Recurrence of microbial keratitis concomitant with antiinflammatory treatment in an animal model. *Cornea.* 1992;11(5):404-408.

58. Gritz DC, Lee TY, Kwitko S, McDonnell PJ. Topical anti-inflammatory agents in an animal model of microbial keratitis. *Archives of ophthalmology.* 1990;108(7):1001-1005.

59. Blair J, Hodge W, Al-Ghamdi S, et al. Comparison of antibiotic-only and antibiotic-steroid combination treatment in corneal ulcer patients: double-blinded randomized clinical trial. *Canadian journal of ophthalmology Journal canadien d'ophtalmologie.* 2011;46(1):40-45.

60. Carmichael TR, Gelfand Y, Welsh NH. Topical steroids in the treatment of central and paracentral corneal ulcers. *The British journal of ophthalmology.* 1990;74(9):528-531.

61. Srinivasan M, Lalitha P, Mahalakshmi R, et al. Corticosteroids for bacterial corneal ulcers. *The British journal of ophthalmology.* 2009;93(2):198-202.

62. Srinivasan M, Mascarenhas J, Rajaraman R, et al. Corticosteroids for bacterial keratitis: the Steroids for Corneal Ulcers Trial (SCUT). *Archives of ophthalmology.* 2012;130(2):143-150.

63. Srinivasan M, Mascarenhas J, Rajaraman R, et al. The steroids for corneal ulcers trial (SCUT): secondary 12-month clinical outcomes of a randomized controlled trial. *American journal of ophthalmology.* 2014;157(2):327-333 e323.

64. Vatansever F, de Melo WC, Avci P, et al. Antimicrobial strategies centered around reactive oxygen species--bactericidal antibiotics, photodynamic therapy, and beyond. *FEMS Microbiol Rev.* 2013;37(6):955-989.

65. Keating A, Pineda R, 2nd, Colby K. Corneal cross linking for keratoconus. *Seminars in ophthalmology.* 2010;25(5-6):249-255.

66. Lamy R, Netto CF, Reis RG, et al. Effects of corneal cross-linking on contrast sensitivity, visual acuity, and corneal topography in patients with keratoconus. *Cornea.* 2013;32(5):591-596.

67. Raiskup-Wolf F, Hoyer A, Spoerl E, Pillunat LE. Collagen crosslinking with riboflavin and ultraviolet-A light in keratoconus: long-term results. *Journal of cataract and refractive surgery.* 2008;34(5):796-801.

68. Vinciguerra P, Albe E, Trazza S, Seiler T, Epstein D. Intraoperative and postoperative effects of corneal collagen cross-linking on progressive keratoconus. *Archives of ophthalmology.* 2009;127(10):1258-1265.

69. Mazzotta C, Hafezi F, Kymionis G, et al. In Vivo Confocal Microscopy after Corneal Collagen Crosslinking. *Ocul Surf.* 2015;13(4):298-314.

70. Sharma N, Suri K, Sehra SV, et al. Collagen cross-linking in keratoconus in Asian eyes: visual, refractive and confocal microscopy outcomes in a prospective randomized controlled trial. *Int Ophthalmol.* 2015;35(6):827-832.

71. Bamdad S, Malekhosseini H, Khosravi A. Ultraviolet A/riboflavin collagen cross-linking for treatment of moderate bacterial corneal ulcers. *Cornea.* 2015;34(4):402-406.

72. Mittal R, Garg P. Re: Said et al.: Collagen cross-linking with photoactivated riboflavin (PACK-CXL) for the treatment of advanced infectious keratitis with corneal melting (Ophthalmology 2014;121:1377-82). *Ophthalmology.* 2014;121(12):e67-68.

73. Uddaraju M, Mascarenhas J, Das MR, et al. Corneal Cross-linking as an Adjuvant Therapy in the Management of Recalcitrant Deep Stromal Fungal Keratitis: A Randomized Trial. *American journal of ophthalmology.* 2015;160(1):131-134 e135.

74. Khan-Lim D, Berry M. Still confused about rose bengal? *Curr Eye Res.* 2004;29(4-5):311-317.

75. Wachter E, Dees C, Harkins J, et al. Topical rose bengal: pre-clinical evaluation of pharmacokinetics and safety. *Lasers Surg Med.* 2003;32(2):101-110.

76. Arboleda A, Miller D, Cabot F, et al. Assessment of rose bengal versus riboflavin photodynamic therapy for inhibition of fungal keratitis isolates. *Am J Ophthalmol.* 2014;158(1):64-70 e62.

77. Amescua G, Arboleda A, Nikpoor N, et al. Rose Bengal Photodynamic Antimicrobial Therapy: A Novel Treatment for Resistant Fusarium Keratitis. *Cornea.* 2017;36(9):1141-1144.

78. Tonogai Y, Ito Y, Iwaida M, Tati M, Ose Y, Hori M. Studies on the toxicity of coal-tar dyes. III. Reason of acute toxicity to fish caused by coal-tar dyes and their industrial effluents. *J Toxicol Sci.* 1980;5(1):23-33.

79. Peng MY, Cevallos V, McLeod SD, Lietman TM, Rose-Nussbaumer J. Bacterial Keratitis: Isolated Organisms and Antibiotic Resistance Patterns in San Francisco. *Cornea.* 2018;37(1):84-87.

80. El Rami H, Chelala E, Dirani A, et al. An Update on the Safety and Efficacy of Corneal Collagen Cross-Linking in Pediatric Keratoconus. *BioMed research international.* 2015;2015:257927.

81. Wittig-Silva C, Chan E, Islam FM, Wu T, Whiting M, Snibson GR. A randomized, controlled trial of corneal collagen cross-linking in progressive keratoconus: three-year results. *Ophthalmology.* 2014;121(4):812-821.

82. Kenward MG, Carpenter J. Multiple imputation: current perspectives. *Statistical methods in medical research.* 2007;16(3):199-218.

83. Ray KJ, Lalitha P, Prajna NV, et al. The Utility of Repeat Culture in Fungal Corneal Ulcer Management: A Secondary Analysis of the MUTT I Randomized Clinical Trial. *Am J Ophthalmol.* 2017.

84. Bhadange Y, Das S, Kasav MK, Sahu SK, Sharma S. Comparison of culture-negative and culture-positive microbial keratitis: cause of culture negativity, clinical features and final outcome. *The British journal of ophthalmology.* 2015;99(11):1498-1502.

85. McLeod SD, Kolahdouz-Isfahani A, Rostamian K, Flowers CW, Lee PP, McDonnell P. The Role of Smears, Cultures, and Antibiotic Sensitivity Testing in the Management of Suspected Infectious Keratitis. *Ophthalmology.* 1996;103(1):23-28.

86. Vemuganti GK, Garg P, Gopinathan U, et al. Evaluation of agent and host factors in progression of mycotic keratitis: A histologic and microbiologic study of 167 corneal buttons. *Ophthalmology.* 2002;109(8):1538-1546.

87. Lineberry N, Berlin JA, Mansi B, et al. Recommendations to improve adverse event reporting in clinical trial publications: a joint pharmaceutical industry/journal editor perspective. *BMJ.* 2016;355:i5078.
